# A Systematic Review of Proteomics in Obesity: Unpacking the Molecular Puzzle

**DOI:** 10.1007/s13679-024-00561-4

**Published:** 2024-05-04

**Authors:** Alba Rodriguez-Muñoz, Hanieh Motahari-Rad, Laura Martin-Chaves, Javier Benitez-Porres, Jorge Rodriguez-Capitan, Andrés Gonzalez-Jimenez, Maria Insenser, Francisco J. Tinahones, Mora Murri

**Affiliations:** 1 Endocrinology and Nutrition UGC, Hospital Universitario Virgen de La Victoria, Málaga, Spain; 2 Instituto de Investigación Biomédica de Málaga y Plataforma en Nanomedicina-IBIMA Plataforma BIONAND, Hospital Clínico Virgen de La Victoria, Málaga, Spain; 3 CIBER Fisiopatología de La Obesidad y Nutrición (CIBEROBN), Instituto de Salud Carlos III, Málaga, Spain; 4 Department of Molecular Genetics, Faculty of Biological Sciences, Tarbiat Modares University, Tehran, Iran; 5 Heart Area, Hospital Universitario Virgen de La Victoria, Instituto de Investigación Biomédica de Málaga y Plataforma en Nanomedicina-IBIMA Plataforma BIONAND, Malaga, Spain; 6 Department of Dermatology and Medicine, Faculty of Medicine, University of Malaga, Malaga, Spain; 7 Department of Human Physiology, Physical Education and Sport, Faculty of Medicine, University of Malaga, Malaga, Spain; 8 Biomedical Research Network Center for Cardiovascular Diseases (CIBERCV), Instituto de Salud Carlos III, 28029 Madrid, Spain; 9 ECAI Bioinformatic Institute of Biomedical Research of Malaga (IBIMA), Malaga, Spain; 10 Diabetes, Obesity and Human Reproduction Research Group, Department of Endocrinology & Nutrition, Hospital Universitario Ramón y Cajal & Universidad de Alcalá & Instituto Ramón y Cajal de Investigación Sanitaria (IRYCIS) & Centro de Investigación Biomédica en Red de Diabetes y Enfermedades Metabólicas Asociadas (CIBERDEM), Madrid, Spain

**Keywords:** Obesity, Proteomics, Metabolism, Personalized medicine

## Abstract

**Purpose of Review:**

The present study aims to review the existing literature to identify pathophysiological proteins in obesity by conducting a systematic review of proteomics studies. Proteomics may reveal the mechanisms of obesity development and clarify the links between obesity and related diseases, improving our comprehension of obesity and its clinical implications.

**Recent Findings:**

Most of the molecular events implicated in obesity development remain incomplete. Proteomics stands as a powerful tool for elucidating the intricate interactions among proteins in the context of obesity. This methodology has the potential to identify proteins involved in pathological processes and to evaluate changes in protein abundance during obesity development, contributing to the identification of early disease predisposition, monitoring the effectiveness of interventions and improving disease management overall. Despite many non-targeted proteomic studies exploring obesity, a comprehensive and up-to-date systematic review of the molecular events implicated in obesity development is lacking. The lack of such a review presents a significant challenge for researchers trying to interpret the existing literature.

**Summary:**

This systematic review was conducted following the PRISMA guidelines and included sixteen human proteomic studies, each of which delineated proteins exhibiting significant alterations in obesity. A total of 41 proteins were reported to be altered in obesity by at least two or more studies. These proteins were involved in metabolic pathways, oxidative stress responses, inflammatory processes, protein folding, coagulation, as well as structure/cytoskeleton. Many of the identified proteomic biomarkers of obesity have also been reported to be dysregulated in obesity-related disease. Among them, seven proteins, which belong to metabolic pathways (aldehyde dehydrogenase and apolipoprotein A1), the chaperone family (albumin, heat shock protein beta 1, protein disulfide-isomerase A3) and oxidative stress and inflammation proteins (catalase and complement C3), could potentially serve as biomarkers for the progression of obesity and the development of comorbidities, contributing to personalized medicine in the field of obesity. Our systematic review in proteomics represents a substantial step forward in unravelling the complexities of protein alterations associated with obesity. It provides valuable insights into the pathophysiological mechanisms underlying obesity, thereby opening avenues for the discovery of potential biomarkers and the development of personalized medicine in obesity

**Supplementary Information:**

The online version contains supplementary material available at 10.1007/s13679-024-00561-4.

## Introduction

Obesity is defined as a nutritional, endocrine and metabolic disorder characterized by an abnormal accumulation of body fat and subclinical chronic inflammation. More than 1 billion people are grappling with obesity [1], which is associated with an increased risk of morbidity and mortality, causing 5 million deaths every year along with overweight [2] https://www.who.int. It is also linked to several chronic diseases such as type 2 diabetes (T2DM), metabolic syndrome, liver disease or cardiovascular diseases and some types of cancer [2].

Body mass index (BMI) is the main parameter used to classify obesity; however, this index does not allow to evaluate body composition because it does not differentiate muscle mass from adipose tissue (AT) or bone [3]. Consequently, it is easy to overestimate obesity and predict wrongly health outcomes. On the one hand, a proportion of individuals with obesity seems to be protected against worsening of metabolic health, whereas at the other end of the spectrum, there are normal weight individuals who have the metabolic abnormalities usually associated with obesity [4]. This paradox hampers new diagnosis and treatment approaches of obesity and all its comorbidities. Additionally, when considering obesity, every person should be assessed based on their own specific and unique circumstances [5]. Therefore, it is essential to find novel molecular parameters that provide a broader understanding of the molecular events that control body weight.

As mentioned earlier, the quality and quantity of body fat accumulation may be responsible for a major risk of developing several pathologies. This is possible due to the fact that adipose tissue (AT) has secretory functions and acts as a metabolic and endocrine organ, capable of producing metabolic regulators [6]. Secreted proteins as well as plasma or serum proteome represent an important group of molecules that provide us valuable insights for monitoring physiological changes caused by obesity [7]. Obesity can alter or even change some proteins in different tissues; an unhealthy amount of body fat caused by protein interactions and modifications are responsible for different affections related to the accumulation of AT. For example, the accumulation of AT around the throat and larynx causes sleep apnoea [8], whereas its accumulation around the heart can lead atrial fibrillation and heart failure [9]. Additionally, obesity can also increase the secretion of certain hormones and pro-inflammatory mechanisms which may produce infertility due to an increase in male hormones, among other issues [10]. The accumulation of fat around various tissues has the potential to induce alterations and modifications at the protein level, as well as in protein–protein interactions.

Proteomics—a powerful approach part of the “OMICS” spectrum—is a promising tool to elucidate the intricate interplay between proteins and obesity. Proteomics can identify and measure changes in protein levels and profiles in response to genetic variations, pathological conditions or physiological states [11]. This technology allows us to investigate how obesity affects cells in different body fluids and tissues such as, visceral adipose tissue (VAT), subcutaneous adipose tissue (SAT), skeletal muscle (SKM), liver, ovarian granulosa cells (GCs), platelets, plasma, sperm, endometrial tissue or extracellular vesicles (EVs).

Obesity, being a multifaceted condition influenced by genetic, environmental, and lifestyle factors, presents a complex disease model. Despite many non-targeted proteomic studies exploring obesity, a comprehensive and up-to-date systematic review of the molecular events implicated in obesity development is lacking. The lack of such a review presents a significant challenge for researchers trying to interpret the existing literature.

Our goal, as the first systematic review of non-targeted proteomics in obesity, is to evaluate all available studies to identify pathophysiological mechanisms that may improve our comprehension of obesity and its clinical implications. We aim to determine which proteins or processes should be prioritized over others while selecting potential targets for hypothesis-driven research. In our review, we have included the latest published research comparing different proteomics profiles of patients with obesity and normoweight subjects. This review provides valuable insights that may contribute to the identification of novel therapeutic targets, and to facilitate the development of new treatments for obesity.

## Methods

### Registration and Protocol

This systematic review was registered a priori at PROSPERO (human studies: CRD42023212429) and structured following the PRISMA guidelines [12].

#### Study Design

We conducted a systematic review of original studies reporting proteomics analysis in general adult human population up to February of 2023. Proteomics analyses were defined as high-throughput analyses conducted at the protein level, aiming to uncover novel insights through non-targeted approaches [13]. Five different electronic databases were searched: PubMed, EMBASE, Scopus, Web of Science (WOS), and Directory of Open Access Journals (DOAJ). Body mass index (BMI) was used to stratify participants as patients with obesity (BMI > 30 kg/m^2^) or normoweight controls (18.5 kg/m^2^ > BMI < 25 kg/m^2^).

### Search Strategy and Data Collection

All the publications that studied the association between proteins with obesity were searched and reviewed. We included studies published using non-targeted proteomics approaches and identifying a characteristic pattern of proteins in patients with obesity compared to healthy normoweight matching controls. In addition, a hand search of the references of the retrieved articles and relevant reviews was performed to identify other potentially eligible studies.

The search algorithm was: “Obesity” [Title] AND “Proteomic” [Title] OR “Proteomics” and the published language was limited to English. We only included studies in humans. Language was limited to English. Experimental methods, protocols, reviews or systematic reviews, abstracts and conference proceedings were excluded from this research. At least two emails with logical intervals (about 3 weeks) were sent to the corresponding author of the manuscript to eliminate the limitations of no access to full text.

All studies were independently screened by three research. Data were extracted independently from included studies by two authors (AR, MI). Disagreements were resolved by discussion between the two authors and consultation was made with a third author (MM). We classified every selected study according to characteristics of the cohort: age, BMI, matching criteria, as it is showed in Table 1. Moreover, analytical, identification or software method used in selected manuscripts, as well as biomarker source and the different abundance of expressed proteins are represented in Table 2.

**Table 1:** Characteristics of published studies included in systematic review comparing samples from patients with obesity and normoweight subjects

**Author (year)**	**Country**	**Simple size (*n*)**		**Age (years)**		**BMI (kg/m**^**2**^**)**		**Matching criteria**
		**Normoweight**	**Obesity**	**Normoweight**	**Obesity**	**Normoweight**	**Obesity**	
**Barrachina et al. 2018**	Spain	22	22	35 ± 11	35 ± 11	22 ± 2	46 ± 5	Age and gender
**Barrachina et al. 2019**	Spain	10	10	34 ± 11	34 ± 12	22 ± 2	46 ± 6	Age and gender
**Benabdelkamel et al. 2015**	Saudi Arabia	7	7	36 ± 5	38 ± 7	23 ± 0	45 ± 4	Age
**Boden et al. 2008**	USA	6	6	36 ± 4	44 ± 4	24 ± 1	34 ± 2	Age
**Giebelstein et al. 2012**	Germany	10	11	51 ± 1	49 ± 1	24 ± 1	34 ± 1	Age
**Giuliani et al. 2022**	USA	6	6	26 ± 9	31 ± 6	22 ± 3	38 ± 6	Age and gender
**Grande et al. 2019**	Italy	4	4	43 ± 4	44 ± 5	22 ± 1	50 ± 1	Age and gender
**Hittel et al. 2005**	USA	6	6	45 ± 3	38 ± 3	24 ± 1	54 ± 4	Gender
**Hwang et al. 2010**	USA	8	8	37 ± 4	44 ± 3	24 ± 1	32 ± 1	Age and gender
**Karlsson et al. 2009**	Sweeden	9	10	47 ± 16	52 ± 19	21 ± 3	33 ± 3	Age and gender
**Kras et al. 2018**	USA	16	17	33 ± 3	32 ± 3	23 ± 1	34 ± 1	Age and gender
**Kriegel et al. 2009**	Germany	5	2	24 ± 4	38 ± 22	22 ± 2	33 ± 3	Gender
**Oberbach et al. 2011**	Germany	15	15	24 ± 2	24 ± 3	24 ± 2	37 ± 7	Age
**Pini et al. 2020**	USA	5	5	38 ± 2	41 ± 2	24 ± 0	33 ± 1	Age
**Shang et al. 2019**	China	8	9	44 ± 7	34 ± 10	22 ± 1	49 ± 10	Gender
**Si et al. 2021**	China	12	14	31 ± 2	38 ± 1	21 ± 0	31 ± 1	Age

Results are shown as mean ± standard deviation (mean ± SD)

**Table 2:** Summary of published studies comparing samples from patients with obesity and normoweight subjects using proteomic approaches

**Author (year)**	**Analytical Method**	**Identification Method**	**Analysis software**	**Sample**	**Protein abundance**
**Barrachina et al. 2018**	2D-DIGE	LC–MS/MS or MALDI-TOF MS	Progenesis SameSpots v 4,5	EVs	22 (9 ↓, 13 ↑)
**Barrachina et al. 2019**	2D-DIGE	LC–MS/MS or MALDI-TOF/TOF	Progenesis SameSpots v 4,5	Platelets	32 (19 ↓, 13 ↑)
**Benabdelkamel et al. 2015**	2D-DIGE	MALDI-TOF MS	Progenesis SameSpots v 3.3	SAT	61 (30 ↓, 31 ↑)
**Boden et al. 2008**	2D-PAGE	MALDI-TOF/TOF	PDQuest	SAT	20 (7 ↓, 13 ↑)
**Giebelstein et al. 2012**	2D-DIGE	nanoHPLC/ESI–MS/MS	DeCyder-2D-V6.5	SKM	26 (12 ↓, 14 ↑)
**Giuliani et al. 2022**	Bis–Tris NuPAGE	nano LC–MS/MS	R package DEP	Endometrial tissue	16 (14 ↓, 2 ↑)
**Grande et al. 2019**	Label free	LC–MS/MS	MaxQuant (MQ) v.1.5.0.30	Platelets	46 (3 ↓, 43 ↑)
**Hittel et al. 2005**	2D-PAGE	MALDI-TOF/TOF MS/MS	Z3 software	SKM	13 (4 ↓, 9 ↑)
**Hwang et al. 2010**	Label free	1D-HPLC–ESI–MS/MS	Affymetrix software	SKM	15 (6 ↓, 9 ↑)
**Karlsson et al. 2009**	2D-PAGE	MALDI-TOF MS	PDQuest	Plasma	11 (6 ↓, 5 ↑)
**Kras et al. 2018**	HPLC–ESI–MS-MS	HPLC–ESI–MS-MS	Proteome software v.4.6.1	SKM	70 (43 ↓, 27 ↑)
**Kriegel et al. 2009**	2D-DIGE	MALDI-TOF/TOF MS/MS	DeCyder software 5.0	Sperm	9 (6 ↓, 3 ↑)
**Oberbach et al. 2011**	2D-DIGE	MALDI-MS/MS Nano-LC-ESI_MS/MS	Delta2D v3.6	Plasma	6 (6 ↑)
**Pini et al. 2020**	Label free	LC–MS	Proteome software v.4.8.9	Sperm	26 (23 ↓, 3 ↑)
**Shang et al. 2019**	Label free	1D-LC–MS/MS	Progenesis software v 4.0	VAT	30 (10 ↓, 20 ↑)
**Si et al. 2021**	TMT	LC/MS	Limma package R 4.1.0	GCs	10 (10↑)

Analytical and identification method of proteins of studies included in systematic review as well as analysis software employed for proteomics analysis is shown. Every expressed protein found showing different abundance are represented in last column as "↑”, increased and “↓”, decreased

*2D*-*DIGE* two-dimensional difference gel electrophoresis, *2D*-*PAGE* two-dimensional polyacrylamide gel electrophoresis, *Bis*–*Tris* NuPAGE protein gel electrophoresis, *HPLC*–*ESI*–*MS*-*MS* high performance liquid chromatography-electrospray tandem mass spectrometry, *TMT* tandem mass Tag, *LC*–*MS*/*MS* liquid chromatography mass spectrometry, *MALDI*-*TOF MS* matrix-assisted laser desorption/ionisation time-of-flight mass spectrometry, *Nano*-*LC*-*ESI*_*MS*/*MS* nano-high performance liquid chromatography/electrospray ionisation mass spectrometry, *1D*-*HPLC*–*ESI*–*MS*/*MS* 1D high performance-liquid chromatography-mass spectrometry, *MALDI*-*MS*/*MS* matrix-assisted laser desorption/ionisation mass spectrometry, *Nano*-*LC*-*ESI*_*MS*/*MS* nano liquid chromatography tandem mass spectrometry, *1D*-*LC*–*MS*/*MS* 1D-liquid chromatography-mass spectrometry, *EVs* extracellular vesicles, *SAT* subcutaneous adipose tissue, *VAT* visceral adipose tissue, *GCs* ovarian granulosa cells, *SKM* skeletal muscle

#### Quality Assessment

The quality assessment of the included studies was assessed independently from included studies by two authors (AR, MI). Disagreements were resolved by discussion between the two authors, and consultation was made with a third author (MM). Study quality was appraised using the Critical Appraisal Skills Programme (CASP) checklists (Online resource 1) in all the included studies of the present systematic review.

## Results

### Characteristics of the Studies

**Fig. 1:**
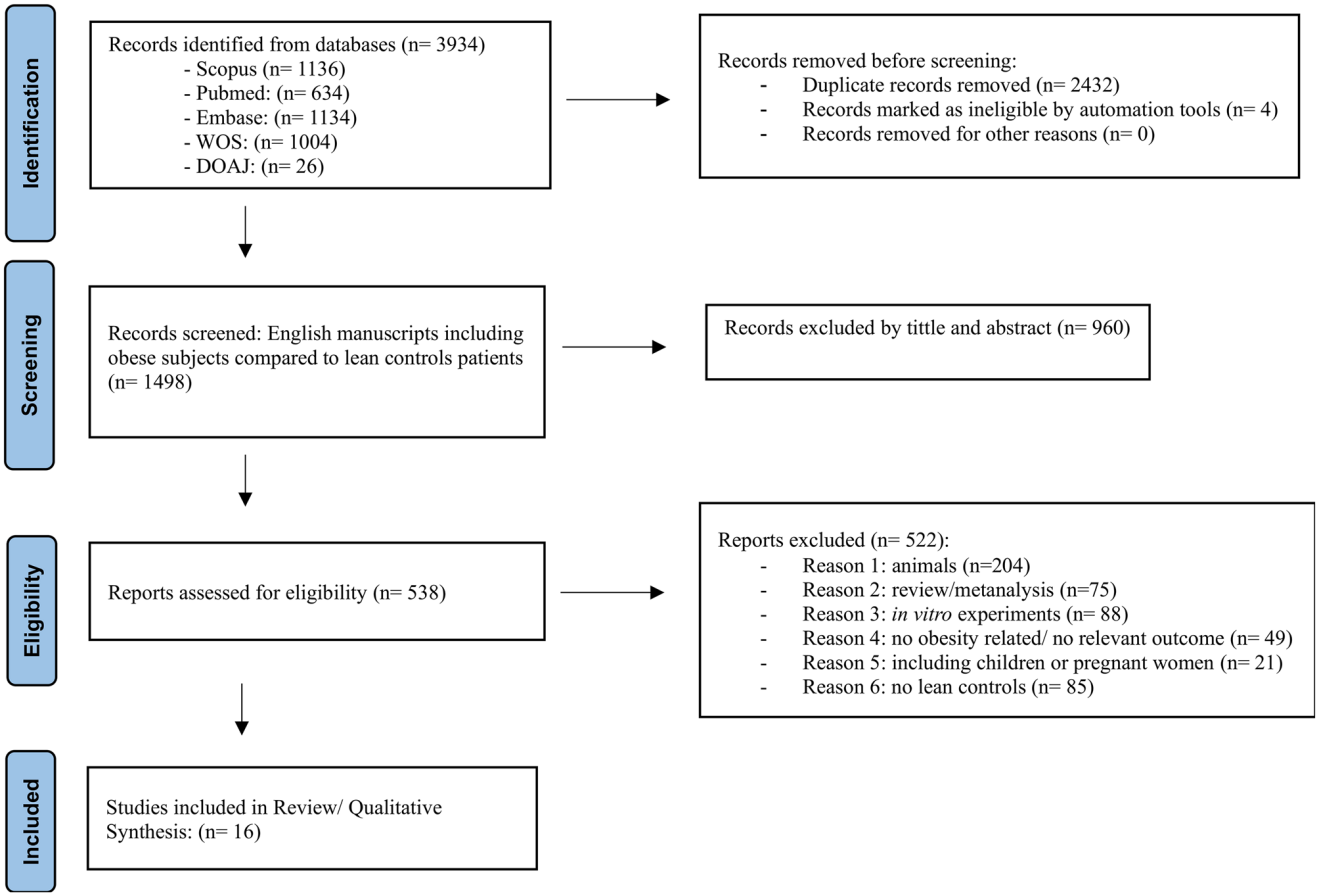
Flow chart for study identification and selection

PRISMA diagram of the individual proteomics systematic search is summarised in Fig. 1. Then, 3934 manuscripts were identified and, according to inclusion criteria, 16 studies were included in systematic review. All articles extracted from mentioned databases were precisely evaluated based on the full text and reported supplementary data.

A summary of the characteristics of the eligible studies for systematic review are summarized in Table 1. The total number of participants included in the current systematic review was 149 normoweight subjects and 152 patients with obesity. The mean age of participants ranged from 24 [14] to 52 years [15]. In 7 studies, control and obese groups were matched by age and gender, 6 studies by age and 3 studies by gender. Different non-targeted proteomics approaches were addressed in order to compare proteins from patients with obesity and lean subject. Examined samples expressing different protein abundance included extracellular vesicles (EVs), platelets, SAT, VAT, SKM, endometrial tissue, GCs and sperm. Analytical and identification methods of the included studies are represented in Table 2. Analytical method included two-dimensional difference gel electrophoresis (2D-DIGE), two-dimensional polyacrylamide gel electrophoresis (2D-PAGE), protein gel electrophoresis NuPAGE Bis–Tris, high performance liquid chromatography-electrospray tandem mass spectrometry (HPLC–ESI–MS-MS), tandem mass tag (TMT) and label free. Identification methods included liquid chromatography mass spectrometry (LC–MS/MS), matrix-assisted laser desorption/ionization time-of-flight mass spectrometry (MALDI-TOF MS), nano-high performance liquid chromatography/electrospray ionisation mass spectrometry (Nano-LC-ESI_MS/MS), 1D-high performance-liquid chromatography-mass spectrometry (1D-HPLC–ESI–MS/MS), matrix-assisted laser desorption/ionization mass spectrometry (MALDI-MS/MS), nano liquid chromatography tandem mass spectrometry (Nano-LC-ESI_MS/MS) and 1D-liquid chromatography-mass spectrometry (1D-LC–MS/MS). Full name of identified proteins along with the corresponding genes can be found in Table 3. UniProt Software was employed to provide comprehensive and functional information of these proteins. Molecular functions, type of analyse sample and protein abundance were reported according to each study.

**Table 3:** Characteristics of proteins found in more than two samples of patients with obesity compared to normoweight subjects in original studies included in the systematic review

**Protein**	**Uniprot number**	**Gen**	**Molecular function**		**Sample**	**Dysregulated proteins**	**Study**
**Actin**	P60709	ACTB	*Polymerizes to produce filaments that form cross-linked networks in the cytoplasm of cells *Cell motility and contraction. Regulate gene transcription and motility and repair of damaged DNA	Structural constituent of cytoskeleton	Platelets	↑	Barrachina et al. 2019
					SKM	↓	Hittle et al. 2005
					Platelets	↓	Grande et al. 2019
**Albumin**	P02768	ALB	*Regulation of the colloidal osmotic pressure of blood *Binds water, Ca2 + , Na + , K + , fatty acids, hormones, bilirubin, and drugs. *Major calcium, magnesium & zinc transporter in plasma	Chaperone binding	Platelets	↑	Barrachina et al. 2019
					SKM	↑	Hittle et al. 2005
					SAT	↑	Benabdelkamel et al. 2015
**Alcohol dehydrogenase class-3**	P11766	ADH5	*Catalyzes the oxidation of long-chain primary alcohols and the oxidation of S-(hydroxymethyl) glutathione * Also oxidises long chain omega-hydroxy fatty acids, such as 20-HETE, producing both the intermediate aldehyde, 20-oxoarachidonate and the product, a dicarboxylic acid	Metabolic pathways	Sperm	↓	Pini et al. 2020
					Platelets	↓	Grande et al. 2019
**Aldehyde dehydrogenase **	P05091	ALDH2	*Catalytic activity: Required for clearance of cellular formaldehyde, a cytotoxic and carcinogenic metabolite that induces DNA damage	Metabolic pathways	SAT	↑	Boden et al. 2008
					SAT	↓	Benabdelkamel et al. 2015
					SKM	↓	Kras et al. 2018
**Alpha enolase**	P06733	ENO1	*Glycolytic enzyme the catalyzes the conversion of 2-phosphoglycerate to phosphoenolpyruvate *Involved in various processes such as growth control, hypoxia tolerance and allergic responses *Stimulates immunoglobulin production	Metabolic pathways	SAT	↓	Benabdelkamel et al. 2015
					SAT	↓	Boden et al. 2008
**Amyloid P-component, serum**	P02743	APCS	*Interacts with DNA and histones and may scavenge nuclear material released from damaged circulating cells *May also function as a calcium-dependent lectin	Inflammation and oxidative stress	EVs	↑	Barrachina et al. 2018
					Plasma	↑	Oberbach et al. 2011
**Annexin A5**	P08758	ANXA5	*Anticoagulant protein that acts as an indirect inhibitor of the thromboplastin-specific complex, which is involved in the blood coagulation cascade	Coagulation cascades	Platelets	↑	Barrachina et al. 2019
					SAT	↑	Benabdelkamel et al. 2015
**Apolipoprotein A-I**	P02647	APOA1	*Participates in the reverse transport of cholesterol from tissues to the liver for excretion *Activates spermatozoa motility	Metabolic pathways	SAT	↓	Benabdelkamel et al. 2015
					Plasma	↓	Karlsson et al. 2009
**Apolipoprotein B-100**	P04114	APOB	*It is a major protein constituent of LDL and VLDL *It functions as a recognition signal for the cellular binding and internalization of LDL particles by the apoB/E receptor	Metabolic pathways	VAT	↑	Shang et al. 2019
					Plasma	↑	Karlsson et al. 2009
**ATP synthase subunit beta**	P06576	ATP5F1B	*Produces ATP from ADP in the presence of a proton gradient across the membrane which is generated by electron transport complexes of the respiratory chain	Metabolic pathways	SAT	↑	Benabdelkamel et al. 2015
					SAT	↑	Boden et al. 2008
**Catalase**	P04040	CAT	*Catalyzes the degradation of hydrogen peroxide (H2O2) to water and oxygen, thereby protecting cells from the toxic effects of hydrogen peroxid *Promotes growth of cells including T-cells, B-cells, myeloid leukemia cells, melanoma cells, mastocytoma cells and normal and transformed fibroblast cells	Inflammation and oxidative stress	SAT	↓	Benabdelkamel et al. 2015
					SKM	↓	Kras et al. 2018
**Coagulation factor V**	P12259	F5	*Central regulator of haemostasis. It serves as a critical cofactor for the prothrombinase activity of factor Xa that results in the activation of prothrombin to thrombin	Coagulation cascades	EVs	↓	Barrachina et al. 2018
					Platelets	↑	Grande et al. 2019
**Complement C3**	P01024	C3	*C3 plays a central role in the activation of the complement system *Acts as a chemoattractant for neutrophils	Inflammation and oxidative stress	EVs	↑	Barrachina et al. 2018
					Plasma	↑	Oberbach et al. 2011
**Creatine Kinase B- type**	P12277	CKB	*Reversibly catalyzes the transfer of phosphate between ATP and various phosphogens *It plays a central role in energy transduction in tissues with large, fluctuating energy demands *Acts as a key regulator of adaptive thermogenesis as part of the futile creatine cycle	Metabolic pathways	SAT	↓	Benabdelkamel et al. 2015
					VAT	↓	Shang et al. 2019
**Crystallin B chain, alpha**	P02511	CRYAB	*May contribute to the transparency and refractive index of the lens. Has chaperone-like activity, preventing aggregation of various proteins under a wide range of stress conditions. In lens epithelial cells, stabilizes the ATP6V1A protein, preventing its degradation by the proteasome (By similarity)	Chaperone binding	SAT	↑	Benabdelkamel et al. 2015
					SAT	↑	Boden et al. 2008
**D-lactate dehydrogenase, mitochondrial**	Q86WU2	LDHD	*Involved in D-lactate catabolic process	Metabolic pathways	VAT	↓	Shang et al. 2019
					SKM	↓	Kras et al. 2018
**Desmin**	P17661	DES	*Essential for proper muscular structure and function *Plays a crucial role in maintaining the structure of sarcomeres, inter-connecting the Z-disks and forming the myofibrils	Structural constituent of cytoskeleton	SKM	↓	Hwang et al. 2010
					SKM	↑	Giebelstein et al. 2012
**Dihydrolipoyllysine residue succinyltransferase component of 2-oxoglutarate dehydrogenase complex, mitochondrial**	P36957	DLST	*Component of the 2-oxoglutarate dehydrogenase complex that catalyzes the overall conversion of 2-oxoglutarate to succinyl-CoA and CO2Catalytic activity	Metabolic pathways	Platelets	↑	Barrachina et al. 2019
					SKM	↓	Kras et al. 2018
					SAT	↓	Boden et al. 2008
**Endoplasmic reticulum chaperone BiP**	P11021	HSPA5	*Endoplasmic reticulum chaperone that plays a key role in protein folding and quality control in the endoplasmic reticulum lumen *Involved in the correct folding of proteins and degradation of misfolded proteins via its interaction with DNAJC10/ERdj5	Chaperone binding	SAT	↑	Benabdelkamel et al. 2015
					Platelets	↓	Grande et al. 2019
**Fibrinogen beta chain**	P02675	FGB	*Mayor function in haemostasis as one of the primary components of blood clots *Stabilize the lesion and guide cell migration during re-epithelialization during the early stages of wound repair	Coagulation cascades	EVs	↑	Barrachina et al. 2018
					Platelets	↑	Barrachina et al. 2019
**Fibrinogen gamma chain**	P02679	FGG	*Mayor function in haemostasis as one of the primary components of blood clots *Stabilize the lesion and guide cell migration during re-epithelialization during the early stages of wound repair	Coagulation cascades	EVs	↑	Barrachina et al. 2018
					SKM	↑	Giebelstein et al. 2012
**Galectin-1**	P09382	LGALS1	*Lectin that binds beta-galactoside and a wide array of complex carbohydrates *Plays a role in regulating apoptosis, cell proliferation and cell differentiation *Inhibits CD45 protein phosphatase activity and therefore the dephosphorylation of Lyn kinase *Strong inducer of T-cell apoptosis	Inflammation and oxidative stress	SAT	↑	Boden et al. 2008
					SAT	↑	Benabdelkamel et al. 2015
**Gamma-synuclein**	O76070	SNCG	*Plays a role in neurofilament network integrity. May be involved in modulating axonal architecture during development and in the adult	Structural constituent of cytoskeleton	SAT	↑	Boden et al. 2008
					VAT	↑	Shang et al. 2019
**Glutathione-S-transferase P**	P09211	GSTP1	*Involved in the formation of glutathione conjugates of both prostaglandin A2 (PGA2) and prostaglandin J2 (PGJ2) *Participates in the formation of novel hepoxilin regioisomers *Regulates negatively CDK5 activity via p25/p35 translocation to prevent neurodegeneration	Inflammation and oxidative stress	SAT	↑	Boden et al. 2008
					Platelets	↓	Grande et al. 2019
**Glyceraldehyde-3-phosphate dehydrogenase**	P0440	GAPDH	*Key enzyme in glycolysis that catalyzes the first step of the pathway by converting d-glyceraldehyde 3-phosphate (G3P) into 3-phospho-d-glyceroyl phosphate *Modulates the organization and assembly of the cytoskeleton	Metabolic pathways	SKM	↑	Hittle et al. 2005
					SKM	↑	Giebelstein et al. 2012
**Heat shock protein beta-1**	P0479	HSPB1	*Small heat shock protein which functions as a molecular chaperone probably maintaining denatured proteins in a folding-competent state *Through its molecular chaperone activity may regulate numerous biological processes including the phosphorylation and the axonal transport of neurofilament proteins *Plays a role in stress resistance and actin organization	Chaperone binding	Platelets	↓	Barrachina et al. 2019
					SAT	↑	Boden et al. 2008
					SAT	↓	Benabdelkamel et al. 2015
**Heat shock protein beta-6**	O14558	HSPB6	*Plays a role in regulating muscle function such as smooth muscle vasorelaxation and cardiac myocyte contractility *May regulate myocardial angiogenesis implicating KDR *Small heat shock protein which functions as a molecular chaperone probably maintaining denatured proteins in a folding-competent state *Plays a role in regulating muscle function such as smooth muscle vasorelaxation and cardiac myocyte contractility	Chaperone binding	SAT	↑	Boden et al. 2008
					SAT	↑	Benabdelkamel et al. 2015
**Haemoglobin subunit alpha**	P6990	HBA1	*Involved in oxygen transport from the lung to the various peripheral tissues	Coagulation cascades	EVs	↓	Barrachina et al. 2018
					SAT	↑	Benabdelkamel et al. 2015
					SKM	↑	Hittle et al. 2005
					SKM	↑	Giebelstein et al. 2012
**Haemoglobin subunit beta**	P68871	HBB	*Involved in oxygen transport from the lung to the various peripheral tissues	Coagulation cascades	EVs	↓	Barrachina et al. 2018
					SAT	↑	Benabdelkamel et al. 2015
**IgGFc-binding protein**	Q9Y6R7	FCGBP	*May be involved in maintenance of the mucosal structure as a gel-like component of the mucosa	Structural constituent of cytoskeleton	EVs	↓	Barrachina et al. 2018
					Endometrial tissue	↑	Giuliani et al. 2022
**Integrin alpha-IIb**	P08514	ITGA2B	*Receptor for fibronectin, fibrinogen, plasminogen, prothrombin, thrombospondin and vitronectin. *Following activation integrin alpha-IIb/beta-3 brings about platelet/platelet interaction through binding of soluble fibrinogen	Coagulation cascades	Platelets	↑	Barrachina et al. 2019
					Platelets	↓	Grande et al. 2019
**Lysozyme C**	P61626	LYZ	*Bacteriolytic function/ catalytic activity; those in tissues and body fluids are associated with the monocyte-macrophage system and enhance the activity of immunoagents *It is capable of both hydrolysis and transglycosylation; it also shows a slight esterase activity. It acts rapidly on both peptide-substituted and unsubstituted peptidoglycan, and slowly on chitin oligosaccharides	Inflammation and oxidative stress	Platelets	↓	Barrachina et al. 2019
					VAT	↑	Shang et al. 2019
					Plasma	↓	Karlsson et al. 2009
**Malate dehydrogenase, cytoplasmic**	P11708	MDH1	*Catalyzes the reduction of aromatic alpha-keto acids in the presence of NADH. Plays essential roles in the malate-aspartate shuttle and the tricarboxylic acid cycle, important in mitochondrial NADH supply for oxidative phosphorylation	Metabolic pathways	Platelets	↓	Grande et al. 2019
					SKM	↓	Giebelstein et al. 2012
**Mitochondrial pyruvate carrier-1**	Q9Y5U8	MPC1	*Mediates the uptake of pyruvate into mitochondria	Metabolic pathways	SKM	↓	Kras et al. 2018
					Sperm	↓	Pini et al. 2020
**Myosin light-chain polypeptide-6**	P60660	MYL6	*Regulatory light chain of myosin. Does not bind calcium	Structural constituent of cytoskeleton	SAT	↑	Boden et al. 2008
					Platelets	↓	Grande et al. 2019
**Parkinson disease protein-7**	Q99497	PARK7	*Multifunctional protein with controversial molecular function which plays an important role in cell protection against oxidative stress and cell death acting as oxidative stress sensor and redox-sensitive chaperone and protease	Inflammation and oxidative stress	SAT	↑	Benabdelkamel et al. 2015
					SKM	↑	Kras et al. 2018
**Protein disulfide-isomerase A3**	P30101	PDIA3	*Catalyzes the formation, isomerization, and reduction or oxidation of disulfide bonds	Chaperone binding	SAT	↓	Boden et al. 2008
					Platelets	↓	Grande et al. 2019
**Phosphatidylinositol 5 phosphate 4 kinase type 2 alpha**	P48426	PIP4K2A	*Catalyzes the phosphorylation of phosphatidylinositol 5-phosphate (PtdIns5P) on the fourth hydroxyl of the myo-inositol ring, to form phosphatidylinositol 4,5-bisphosphate. Has both ATP- and GTP-dependent kinase activities	Metabolic pathways	Platelets	↓	Barrachina et al. 2019
					Platelets	↓	Grande et al. 2019
**Pyruvate kinase**	P30613	PKM	*Catalyzes the final rate-limiting step of glycolysis by mediating the transfer of a phosphoryl group from phosphoenolpyruvate (PEP) to ADP, generating ATP	Metabolic pathways	SKM	↑	Kras et al. 2018
					SKM	↓	Hittle et al. 2005
					Platelets	↓	Grande et al. 2019
**Tropomyosin alpha-1 chain**	P09493	TPM1	*Binds to actin filaments in muscle and non-muscle cells. Plays a central role, in association with the troponin complex, in the calcium dependent regulation of vertebrate striated muscle contraction	Structural constituent of cytoskeleton	Platelets	↑	Barrachina et al. 2019
					Platelets	↓	Grande et al. 2019
**Vimentin**	P08670	VIM	*Class-III intermediate filaments found in various non-epithelial cells, especially mesenchymal cells *Vimentin is attached to the nucleus, endoplasmic reticulum, and mitochondria, either laterally or terminally	Structural constituent of cytoskeleton	SAT	↑	Boden et al. 2008

### A Proteomics Approach to Obesity

All outcome data were semi-quantitative, i.e. relative protein abundance. In total, the abundance of 362 proteins was reported to be statistically different between individuals with obesity compared to normoweight controls. Forty-one proteins were found to be altered in at least 2 studies (Table 3) and were described in detail according to their function in this section. As those proteins were not reported in the same kind of samples in at least 3 different studies, a meta-analysis was not performed.

#### Proteins Related to Metabolic Pathways

Obesity involves a sustained abundance of available energy; therefore, metabolic pathways that play an important role in maintaining cellular energy homeostasis are often dysregulated under this condition [16]. The excess of energy intake associated with the progression of obesity can result in hyperglycaemia, hyperinsulinemia and an increasement in fat mass. Moreover, the expansion of AT requires the formation of new blood vessels that supply nutrients and oxygen to proliferate adipocyte cells. Multiple proteins orchestrate the processes required for the efficient production of this energy demand [17]. In this regard, 7 proteins related to metabolic pathways have shown a consistent decrease in subjects with obesity as compared to their normal-weight counterparts, while 3 proteins were found to be increased and 3 proteins were found increased/decreased in different studies.

##### Alcohol Dehydrogenase Class-3

Alcohol dehydrogenase class-3 (ADH5) possesses a great ability to metabolize long-chain alcohols, playing a significant role in the metabolism of formaldehyde in the human body. They have attracted considerable interest due to its detoxifying role, modulating the effects of ingested ethanol, tissue damage and developmental abnormalities [18]. A previous study in obesity has shown that ADH5 transcript is significantly increased in brown adipose tissue (BAT) from patients with obesity, suggesting its role in protecting BAT against obesity-associated metabolic dysfunction [19]. Moreover, differential expression of ADH5 abundance during gestation and lactation has been correlated with weight gain in early life [20]. Decreased abundance of ADH5 together with some other proteins in obesity compared to normoweight patients was related to several biological processes, including oxidative stress, inflammation, translation, DNA damage repair and sperm function, being significantly less abundant in the sperm of men with obesity compared with healthy weight controls [21]. Low protein abundance of ADH5, thus, suggest that oxidative stress together with other biological process as inflammation are closely tied to reproductive dysfunction in men with obesity, compromising their fertility [21]. Additionally, patients with obesity have reduced abundance of ADH5 in platelets [22].

##### Aldehyde Dehydrogenase 

Aldehyde dehydrogenase (ALDH2) is an important enzyme that eliminates toxic aldehydes by catalysing their oxidation to non-reactive acids. There is a consistent association with ALDH2 and obesity-related features including BMI, waist circumference, waist-to-hip ratio, and visceral fat accumulation [23]. Boden et al. revealed an increase of ALDH2 on SAT of patients with obesity and IR [24]. Those results differ from Benabdelkamel et al. who reported a decrease in abundance of this antioxidant enzyme on SAT [25]. Low abundance of ALDH2 is known to lead accumulation of toxic acetaldehyde and lipid aldehydes as well as to decrease in lipolysis within the mature adipocytes of SAT; however, these reported differences could be explained by the difference of proteomics analysis employed, more recent in the case of Benabdelkamel et al. [25] and the significative differences in BMI of population with obesity from both studies [24, 25]. Boden et al. employed subcutaneous fat biopsies from the upper thighs of six nondiabetic subjects with obesity while Benabdelkamel et al. employed SAT obtained by liposuction from patients with morbid obesity [24, 25]. On the other side, a decreased ALDH2 abundance was also found in mitochondria isolated from SKM of individuals with obesity compared to lean healthy controls [26], highlighting how obesity can alter the expression of mitochondrial proteins regulating key metabolic processes in SKM, including ATP production or fatty acid oxidation [27, 28].

##### Alpha-Enolase

Alpha-enolase (ENO1) is a glycolytic enzyme involved in various processes such as energy or free fatty acid metabolism, growth control, and hypoxia tolerance [29]. Previous studies in obesity have reported decreased levels of ENO1 in adults with obesity and T2DM. It proves that inhibiting the non-glycolytic functions of alpha-enolase can generate an antidiabetic effect and weight loss in those individuals; however, it is needed further research [29]. In this regard, ENO1 downregulation have been associated with weight regain in patients with obesity who followed a weight loss program, indicating the role of ENO1 expression changes on finding target for preventing weight regain and treat metabolic disorders [30]. These findings are consistent with decreased levels of ENO1 abundance found on SAT of patients with obesity in two of the original manuscripts included in the present systematic review. A significant decrease in the abundance of this enzyme was also reported by Benabdelkamel et al., highlighting alterations in energy metabolism, including a decrease in the glycolytic activity due to weight gain in obesity [25].

##### Apolipoprotein A-I

Apolipoprotein A-I (APOA1) is the major peptide of human plasma high-density lipoproteins (HDL), which are crucial for reverse cholesterol transport from tissues to the liver excretion. This apolipoprotein has anti-inflammatory, antiatherogenic and anti-thrombotic properties, interacting with HDL particles and giving them their cardioprotective characteristics. Moreover, previously, it has been reported that plasma levels of APOA1 are inversely associated with some metabolic conditions, including T2DM, hyperlipidaemia, NAFLD and obesity [31]. Some other reported lipoprotein abnormalities, including decreased levels of APOA1 in patients with obesity compared to lean individuals [32]. In this regard, APOA1 abundance was found to be decreased in plasma from patients with obesity compared to healthy control individuals [15]. Those new findings are consistent with Benabdelkamel et al. who found significantly decreased APOA1 abundance on SAT of patients with morbid obesity compared to participants with overweight or lean controls [25]. Moreover, LDL proteome of women with obesity showed higher levels of APOA1 than men with obesity, highlighting sex-related differences [15].

##### Apolipoprotein B-100

Apolipoprotein B-100 (APOB) is a major protein constituent of LDL. Increased levels of this molecule are well established to be solid predictors for cardiometabolic events [33]. In this regard, recent studies reported that higher levels of APOB were a good risk predictor for long-term cardiovascular events in patients with obesity [34]. Moreover, increased abundance of this protein in patients with obesity were decreased by bariatric surgery, revealing the role of this intervention in reducing levels of APOB and decreasing risk of cardiometabolic diseases [35]. In addition, increased levels of APOB were found in plasma and VAT from patients with obesity compared to normoweight individuals revealing it concerning and important role in cardiometabolic health outcomes of patients with obesity [15, 36].

##### ATP Synthase Subunit Beta

ATP synthase subunit beta (ATP5F1B) is a target enzyme for human health that produces ATP from ADP in the presence of a proton gradient across the membrane which is generated by electron transport complexes of the respiratory chain. Malfunction of this complex has been implicated in a wide variety metabolic disease. ATP5F1B plays a beneficial role in obesity-induced non-alcoholic fatty liver disease (NAFLD) by improving mitochondrial function in hepatic steatosis [37]. Inhibition of this enzyme could alter energy and lipid metabolism, contributing to an insulin resistant phenotype and to the onset and progression of T2DM [38]. In addition, an impaired ATP5F1B translation has been correlated with the suppression of muscle metabolism in SKM of individuals with obesity compared to lean subject [39]. ATP5F1B abundance was increased in SAT samples from insulin-resistant patients with obesity compared to lean insulin-sensitive controls [40], demonstrating an important role in the development of obesity-related IR and inflammation due its involvement in energy and free fatty acid metabolism. In accordance with this result, ATP5F1B was also increased on SAT from individuals with morbid obesity compared to lean subjects, supporting those metabolic differences in both groups [25].

##### Creatine Kinase B-Type

Creatine kinase B-type (CKB) is a cytoplasmic enzyme involved in energy homeostasis. It reversibly catalyses the transfer of phosphate between ATP and various phosphagens, playing a central role in energy transduction in tissues with large and fluctuating energy demands as AT [41]. Moreover, CKB abundance is strongly induced by thermogenic stimulation in adipocytes. In this regard, recent studies in obesity reported that inactivation of CKB in adipocytes decrease thermogenic capacity, highlighting the important role of BAT in energy expenditure by generating heat through this process [42]. Decreased levels of CKB abundance were found on SAT and VAT of individuals with obesity compared to lean subjects [25, 43]. Shang et al. revealed a downregulation of CKB in VAT of women with morbid obesity who underwent bariatric surgery compared to normoweight females who underwent elective abdominal surgical procedures [43]. Those results are consistent with Benabdelkamel et al. who also reported a decreased abundance of CKB on SAT from patients with morbid obesity, but also in individuals with overweight compared to lean controls [25].

##### Dihydrolipoyllysine-Residue Succinyltransferase

Dihydrolipoyllysine-residue succinyltransferase (DLST) is a component of one of the rate-limiting enzyme complexes in the tricarboxylic acid cycle, playing a role in carbohydrate metabolism [44]. DLST has been reported to contribute to energy expenditure by enhancing the mitochondrial lipoylation pathway [45]. In this regard, DLST protein abundance was found increased in platelets of individuals with obesity compared to controls [46], while a decreased abundance of this protein was reported in subjects with obesity by Kras et al. and Boden et al*.* in SAT and SKM [24, 26].

##### d-Lactate Dehydrogenase

d-lactate dehydrogenase (LDHD) is a mitochondrial protein that catalyses specifically the reduction of d-lactate to pyruvate with concomitant oxidation of NAD^+^ to NADH [47]; it may play a role in regulating apoptosis, cell proliferation and cell differentiation, serving as a general indicator of acute and chronic diseases [48]; however, there is not much evidence available associating altered abundance of this protein in individuals with obesity. In contrast, LDHD was found decreased in VAT and SKM from individuals with obesity [26, 43]. These findings, thus, suggest that downregulation of this mitochondrial protein could have a potential role as a biomarker in obesity and its associated metabolic diseases.

##### Glyceraldehyde-3-Phosphate Dehydrogenase

Glyceraldehyde-3-phosphate dehydrogenase (GAPDH) modulates the organization and assembly of the cytoskeleton. It is one of the central enzymes in glycolysis, generating NADH, which is a source of energy and metabolites for several diseases [49]. Altered GAPDH expression in human muscle has been associated with IR and a tendency towards a higher lipogenic gene expression, which are associated with obesity phenotype [50]. In addition, significant increased abundance of glycolytic enzymes, including GAPDH, were found in women with obesity or morbid obesity, relative to lean control subjects. It was reported an increased protein abundance of GAPDH in SKM of individuals with obesity compared to lean patients [51, 52]. These alterations may balance the progressive decrease in muscle mitochondrial function of individuals with obesity, contributing to the loss of glucose and lipid homeostasis over time, and to the eventual development of obesity-related diseases such as T2DM. In contrast, a low abundance of GAPDH was found in platelets of individuals with obesity compared to lean patients [22]; however, limitations of the study, including number and gender of individuals analysed should be considered.

##### Malate Dehydrogenase, Cytoplasmic

Malate dehydrogenase (MDH1) catalyses the interconversion of malate and oxaloacetate in the mitochondrial membrane, playing an indispensable role in ATP generation [53]. It represents a source of energy in differentiated cells, supporting proliferation and glucose consumption, but also acting as a prognostic biomarker in some life-threating situations [53]. Previous studies in obesity reported that acetylation levels of MDH1 are significantly increased during adipocyte differentiation [53, 54]. Adipocyte differentiation can contribute to the development of obesity via a positive energy balance. Interestingly, increased levels of MDH1 in adipocytes induce enhanced adipogenesis in these cells, increasing its enzymatic activity [54]. These findings contrast with Grande et al. and Giebelstein et al. who reported decreased abundance of this enzyme in platelets and SKM of individuals with obesity [52, 55]. In this line, decreased levels of MDH1 are related to metabolic disorder of the malate–aspartate shuttle as well as disruption in several pathways, which may indicate that the decrease in MDH1 and subsequent reduction in the NAD/NADH is a likely mechanism of cellular aging controlled by carbohydrate metabolism [52, 55].

##### Mitochondrial Pyruvate Carrier-1

Mitochondrial pyruvate carrier-1 (MPC1) is a key metabolic protein that regulates the transport of pyruvate into the mitochondrial inner membrane [56]. It is critical for several major biological pathways of carbohydrate, lipid, and amino acid metabolism, providing energy [57]. Moreover, it has emerged as a promising pharmacological target for metabolic disorders by modulating mitochondrial function [58]. MPC1 abundance was found decreased in several kind of samples of individuals with obesity, including SKM or sperm [21, 26], suggesting that a decrease in MPC1 may promote dysfunctional mitochondria metabolism in obesity.

##### Phosphatidylinositol 5-Phosphate 4-Kinase Type-2 Alpha

Phosphatidylinositol 5-phosphate 4-kinase type-2 alpha (PIP4K2A) is a protein implicated in lipid metabolism, including various aspects of intracellular cholesterol transport [59]. Barrachina et al. and Grande et al. both identified decreased abundance of PIP4K2A in platelets of individuals with obesity compared to normoweight controls [22, 46]. Platelets are key players in the pathophysiology of several diseases related to obesity [60], and their function and size have been reported to be altered in obesity [61]. Decreased abundance of PIP4K2A in these cells suggests that PIP4K2A could be responsible at least in part for the platelet alterations observed in obesity [46].

##### Pyruvate Kinase

Pyruvate kinase (PKM) is an enzyme that catalyses the conversion of phosphoenolpyruvate and ADP to pyruvate and ATP in glycolysis. It plays an essential role in regulating cell metabolism [62]. Recent evidence suggests the involvement of this enzyme modulators in several metabolic diseases. PKM may regulates gene activation in the context of inflammation and metabolic reprogramming, being a promising target for addressing some pathologies, including obesity [63]. An increased abundance of this metabolic protein was reported by Kras et al. in SKM of individuals with obesity [26]. However, a previous proteomics study in obesity reported decreased levels of PKM in the same tissue of individuals with obesity compared to lean controls [51]. This could be explained by differences in muscle location of patients. Hittle et al. reported a decreased abundance of PKM in SKM [51], while Kras et al. reported an increased abundance of this protein in collected biopsies from the vastus lateralis muscle [26]. In addition, Grande et al. reported decreased levels of PKM in platelets of patients with obesity [55].

##### Proteins Related to Chaperon Binding

Proteins that facilitate the folding of other proteins are called chaperones. Chaperones are a broad class of proteins that maintain protein homeostasis by monitoring the quality and integrity of protein structure. They have the potential to prevent nonspecific aggregation by binding to non-native proteins and assist in protein folding [64]. Protein folding is a process that usually takes place in the ER, eliciting proper forming of proteins [65]. Given the growing body of research indicating the involvement of ER stress in various disease pathologies, including obesity, the concept of enhancing ER folding capacity through chemical chaperones has emerged as a promising therapeutic strategy, especially in the context of metabolic disorders [66]. Most of chaperon binding proteins abundance have been found to be increased in individuals with obesity compared to normoweight matching controls (Table 3) except for 2, that have been found increased/decreased in different studies and 1 that have shown a consistent decrease.

##### Albumin

Albumin (ALB) is the most abundant extracellular chaperone protein. ALB is the most significant modulator of colloid osmotic-pressure and transports a large variety of molecules such as fatty acids, drugs or hormones. Moreover, it acts as an antioxidant agent [67]. Values of serum ALB has been reported to be decreased in obesity. This state of inflammation may lead to altered ALB levels among the population with obesity compared to healthy controls, making this condition an independent predictor of hypoalbuminemia [68]. In addition, ALB concentration was positively associated with the prevalence of metabolic syndrome (MetS), whereas an increase in ALB over time might protect against MetS development [67]. ALB abundance was found increased in several samples including, platelets, SAT or SKM proteome of individuals with obesity compared to normoweight matching controls. Differentially altered ALB abundance thus, may point to increase according to BMI [25, 46, 51].

##### Crystallin B Chain, Alpha

Crystallin B chain alpha (CRYAB) is one of the most widespread and represented of the human small HSP. CRYAB prevents aggregation of various proteins under a wide range of stress conditions. It is greatly expressed in tissues with high rates of oxidative metabolism, such as skeletal and cardiac muscles [69]. Dysfunctions of this protein are associated with several metabolic diseases due to its important role in protection of muscle tissues from the alterations of protein stability [69]. Increased levels of CRYAB in the obesity as well as in weight regain after long-term weight loss maintenance have been demonstrated previously [30, 70], supporting the role of CRYAB in obese phenotype. These results are consistent with our findings. Benabdelkamel et al. and Boden et al. revealed high abundance rates of CRYAB on SAT of individuals with obesity compared to normoweight controls [24, 25]. This could influence weight management, revealing CRYAB as a biomarker of obesity and a mediator of weight control.

##### Endoplasmic Reticulum Chaperone BiP

Endoplasmic reticulum chaperone BiP (HSPA5) is a molecular chaperone involved in the correct folding and assembly of proteins, and in the degradation of misfolded proteins in the endoplasmic reticulum (ER). HSPA5 is a master regulator of ER homeostasis and functions, and it is an essential component of the protein translocation machinery into the endoplasmic reticulum (ER) [71]. Previous studies have reported that HSPA5 abundance was increased in AT of patients with obesity highlighting its direct association between BMI and other metabolic factors including IR or hypertriglyceridemia [72]. In this regard, Benabdelkamel et al. reported higher abundance of HSPA5 on SAT of individuals with obesity compared to lean controls [25]. On the other hand, Grande et al. identified a lower abundance of HSPA5 in platelets of individuals with obesity compared to lean subjects [55]. Those differences in protein abundance may indicate that HSPA5 role in obesity need to be further investigated, however, limitations of studies, including small number of individuals analysed or sex- related differences should be considered [25, 55].

##### Heat Shock Protein Beta 1

Heat shock protein beta 1 (HSPB1) is a ubiquitous chaperone involved in key physiological and cellular pathways such as inflammation, immunity or apoptosis [73]. It also mediates the survivability of the cells under various stressful conditions, as it is able to control the redox state of the cell [74]. Previous studies in obesity showed a direct association between HSPB1 abundance and BMI, or high levels of HSPB1 and IR, suggesting an important role in metabolic disorders [75]. In addition, Boden et al. reported a higher HSPB1 abundance in SAT proteome of individuals with obesity compared to lean controls, supporting those previous findings [24]. However, results of Barrachina et al. and Benabdelkamel et al. reported a downregulation of this protein in platelets and SAT of patients with obesity, respectively [25, 46].

##### Heat Shock Protein Beta 6

Heat shock protein beta-6 (HSPB6) plays an essential role as molecular chaperones in proteostasis and cell growth and survival [74]. Heat shock proteins (HSP) are produced in response to multiple stressors [76]. HSPB6 is the most upregulated HSP protein during differentiation of human adipose-derived stem cells into mature adipocytes [77] and it has been reported to be a negative regulator of adipocyte function [78]. Benabdelkamel et al. and Boden et al. revealed an increased abundance of HSPB6 on SAT of individuals with obesity [24, 25].

##### Protein Disulfide-Isomerase A3

Protein disulfide isomerase A3 (PDIA3) is a chaperone protein that modulates protein folding of newly synthesized glycoproteins and responds to endoplasmic reticulum (ER) stress [79]. Previous studies in obesity showed that circulating levels of PDIA3 were increased in pediatric subjects with obesity compared to controls. PDIA3 circulating levels were positively associated with obesity markers, IR and LDL-cholesterol. This is evidence that PDIA3 could be an early marker of IR, dyslipidemia and other obesity-related complications [80]. Those results match similar studies in adult population, emphasizing the role of PDIA3 in obesity [81]. Moreover, downregulation of PDIA3 abundance was also reported in two independent proteomics analyses selected for the present systematic review, including samples of SAT and platelets in individuals with obesity compared to lean patients [24, 55].

##### Proteins Related to Coagulation Cascades

Obesity is characterized by the elevation of several clotting factors and PAI-1 directly affecting coagulation [82]. The involvement of adipose tissue to increase the thrombotic tendency has been proposed through several mechanisms involving platelet function abnormalities and increased coagulation, together with endothelial dysfunction [82, 83]. No proteins related to coagulation cascades have shown a consistent decrease in subjects with obesity as compared to their normal-weight counterparts, while 3 proteins were found to be increased and 4 proteins were found increased/decreased in different studies as it is reported in Table 3. This may suggest that alterations in abundance of coagulation cascades proteins have an important role in obesity as they can modulate several metabolic disorders [84].

##### Annexin A5

Annexin A5 (ANXA5) is the most abundant annexin and it is expressed ubiquitously. It has an anticoagulant function and a potential role in cellular signal transduction, inflammation, and differentiation [85]. Moreover, it appears to play a role in triglyceride metabolism [86]. ANXA5 protein was induced in adipocytes during aging [87] and one study demonstrated an association of ANXA5 polymorphisms with obesity in a Korean patient cohort [88], which may suggest a function of ANXA5 on the fat deposition, storage or mobilization. Barrachina et al. and Benabdelkamel et al. reported increased levels of ANXA5 in platelets and SAT of individuals with obesity compared to lean healthy controls [25, 46]. As it is stated, higher abundance of ANXA5 in proteome of individuals with obesity may be associated with the development of obesity and could mediate in some coagulation disorders related to this condition [88].

##### Coagulation Factor V

Coagulation factor V (F5) is the central regulator of haemostasis. It plays an important role in the propagation phase of coagulation as a component of the prothrombinase complex [89]; however, it mediates both procoagulant and anticoagulant functions as a result of the activity of proteases [90]. Alterations in coagulation have been widely studied in obesity. A recent study comparing individuals with obesity and normoweight controls showed that BMI contributes to hypercoagulability, highlighting those individuals with obesity are more hypercoagulable [91]. F5 activity was found to be statistically insignificant in plasma of patients with obesity compared to control [92], which is consistent with Barrachina et al. [46]. They both reported decreased abundance of this protein in plasma of individuals with obesity, highlighting its role in cardiovascular events in obesity [46]. However, Grande et al. showed that F5 was increased in platelets from patients with obesity compared to non-obese controls [55].

##### Fibrinogen

Fibrinogen plays key roles in both blood clotting and platelet aggregation. Fibrinogen is a hexameric plasmatic glycoprotein composed of pairs of three chains: fibrinogen alpha chain, fibrinogen beta chain (FGB) and fibrinogen gamma chain (FGG). The most significant biological role of fibrinogen is related to its ability to form the scaffold of a blood clot and prevent the loss of blood after injury [93]. However, fibrinogen together with fibrin plays important and overlapping roles in fibrinolysis, cellular and matrix interactions and inflammation [93]. Several studies have reported higher plasma fibrinogen levels in subjects with obesity. More specifically, FGG has been found increased in women and children with obesity [94]. Protein abundance of FB and FGG were reported to be increased in several tissues, including EVs, platelets and SKM, of patients with obesity compared to lean individuals in three independent studies [52, 95].

##### Haemoglobin

Haemoglobin (Hb) is an iron-containing metalloprotein that transports oxygen molecules from the lungs to the rest of the human body. Haemoglobin consists of protein subunits haemoglobin subunit alpha 1 (HBA1) and alpha 2 (HBA2), and haemoglobin subunit beta (HBB) [96]. Circulatory Hb levels have been reported to be increased in patients with obesity [97]; however, another study reported not differences [98]. In this regard, HBA1 and HBB were found to be decreased in serum proteome of metabolically abnormal individuals with obesity compared to controls [99]. However, different abundance of both subunit proteins was variable depending on the analysed sample selected. Barrachina et al*.* reported a decrease of HBA1 and HBB in EVs of women with obesity compared to normoweight controls [46], while an increase of both proteins was found on SAT and SKM in three other independent studies [25, 51, 52].

##### Integrin Alpha-IIb

Integrin alpha-IIb (ITGA2B) is a highly abundant heterodimeric platelet receptor that can transmit information bidirectionally across the plasma membrane and plays a critical role in haemostasis and thrombosis and platelet aggregation [100]. ITGA2B levels were reported to be reduced in platelet particles from subjects with obesity using flow cytometry [55]. Two studies of the present systematic review found contradictory ITGA2B protein abundance results in obesity. Grande et al. reported a decrease of ITGA2B abundance in platelets of woman with obesity compared to lean matching controls [55]. However, Barrachina et al. found that ITGA2B was increased in patients with obesity compared to lean healthy controls [46]. These contradictory results may be a result of the effect of sex.

##### Proteins Related to Structure/Cytoskeleton

Seven proteins related to structure/ cytoskeleton have shown altered abundance in subjects with obesity as compared to their normal-weight matching controls. Two of them have shown a consistent increase, while 5 proteins were found increased/decreased in different studies. Any of the analysed studies reported a consistent decrease of these types of proteins. This suggests the significant role in adipose tissue growth of proteins related to structure/ cytoskeleton as they are involved in both hypertrophy and hyperplasia of fat cells.

##### Actin, Cytoplasmic 1

Actin (ACTB) is the most abundantly expressed protein in eukaryotic cells and is the key building block of the filamentous actin cytoskeleton. It is an essential component for almost all actin-dependent cellular processes, including cell migration, cell cycle progression, chromatin remodelling and gene expression and DNA damage response [101]. ACTB has been used as a reference protein/gene in many studies including those for obesity studies [102]. However, proteomics studies reported in the present systematic review found altered abundance of ACTB protein in obesity. While Barrachina et al. reported an increase in ACTB abundance in subjects with obesitycompared to lean individuals, Grande et al. and Hittle et al. reported a decreased ACTB abundance in platelets and SKM of patients with obesity, respectively [22, 46, 51].

##### Desmin

Desmin (DES) is a muscle-specific intermediate filament protein and a key subunit of the intermediate filament in cardiac, skeletal, and smooth muscles [103]. It plays a critical role in the maintenance of sarcomeres structures, forming the myofibrils, and in mechanical integrity of the contractile apparatus in muscle tissues [103]. Previous studies showed that DES deletion is associated with mitochondrial dysfunction in muscle cells, which may result in altered metabolism and therefore, alter muscle function [104]. Moreover, DES has a strong association with the development of some cardiometabolic diseases, including obesity [56, 105]. Giebelstein et al. reported increased expression of DES in SKM of individuals with obesity compared to lean controls [52]. However, a previous proteomics study describing differences in proteins abundance related to obesity in the same tissue revealed decreased abundance of DES in individuals with obesity compared to lean controls [106].

##### Gamma-Synuclein

Gamma-synuclein (SNCG) is an adipocyte-neuron gene with several implications in metabolic health due to its high abundance in white adipose tissue (WAT) [107]. In addition, SNCG plays an important role in adipocyte physiology. Previous studies in obesity reported that SNCG is highly expressed in human WAT and increased in obesity. Oort Pieter et al. reported that upregulation of SNCG is nutritionally regulated in WAT whereas its loss partially protects from high-fat diet induced obesity and ameliorates some of the associated metabolic complications, highlighting the role of SNCG overexpression in obesity [107]. In this regard, decreased levels of this protein in human have been correlated with protection against obesity following high fat diet [108]. Those findings are consistent with Boden et al. and Shang et al., who described an increased abundance of SNCG in both SAT and VAT of individuals with obesity compared to lean controls [24, 43].

##### IgGFc-Binding Protein

IgGFc-binding protein (FCGBP) is one of the core mucus proteins which are produced and secreted by goblet cells, which has an essential role in protection of epithelial surfaces or mucosal defence. A recent meta-nalysis reporting AT epigenetic profile in obesity showed that FCGBP gene is hypomethylated in metabolically unhealthy individuals with overweight or obesity [96]. This protein is expressed in mucin secreting cells in tissues such as the colon, small intestine or gall bladder, providing an anti-inflammatory function which may confer some protection against the obesity induced low-grade inflammatory state [96]. FCGBP protein abundance was found increased in endometrial tissue of women with obesity compared to normoweight matching controls but decreased in EVs of a similar population of women with obesity [46, 109].

##### Myosin Light-Chain Polypeptide-6

Myosin light-chain polypeptide-6 (MYL6) is a hexameric ATPase cellular motor protein. It mediates airway smooth muscle contractile function, which is related to asthma due to the excessive airway narrowing produced by an increase in contractility of this tissue. This contractile response is reported to be enhanced in obesity [110]. MYL6 has been associated to metabolic alterations [111]. Previous studies in animals reported an upregulation of MYL6 in lung tissue of obese mice compared to lean control after a nutritional intervention based on a high fat diet [112]. Moreover, higher differences in MYL6 gene expression were found in placenta of pregnant women with obesity compared to lean matching controls [113]. In the present review, it was found that Boden et al. reported an increased abundance of MYL6 on SAT of individuals with obesity [24], while Grande et al. described a decreased abundance of MYL6 in platelets of women with obesity compared to lean controls [55]. Differences in MYL6 abundance may be explained by the tissue sample or by the gender analysed.

##### Tropomyosin Alpha-1 Chain

Tropomyosin alpha-1 chain (TPM1) is an actin-binding protein involved in the contractile system of striated and smooth muscles and the cytoskeleton of non-muscle cells. Mutations in this gene have been associated with physio pathological process related to several cardiometabolic diseases, such as obesity [114]. A recent study reported that expression level of TPM1 was decreased in subjects with obesity after a high-fat diet, while it was increased after an antioxidant intervention due to a reduction in process mediated by oxidative stress [115]. Therefore, it was suggested that obesity may damage the structure and function of the heart by down-regulating TPM1 expression. Conflicting proteomics results have been reported, while Grande et al. reported a decrease of TPM1 abundance in platelets of individuals with obesity compared to lean controls [55], Barrachina et al. reported an increase of TPM1 abundance in platelets of subjects with obesity[46].

##### Vimentin

Vimentin (VIM) is an abundant cytoplasmic protein which is recognized for its important role in stabilizing intracellular structure and its mechanical role in cell plasticity and stress absorbers [116]. VIM is expressed in mesenchymal origin, including adipocytes where it forms lipid droplets and stabilizes triglycerides [117]. Moreover, VIM participates in lipolysis through direct interactions with hormone-sensitive lipase [118]. In this regard, previous studies reported that a lack of VIM results in less fat accumulation [119]. In addition, it has been pointed out that VIM deficiency prevents high-fat diet-induced obesity [120]. Those findings are in the same direction of proteomics study results. Boden et al. and Benabdelkamel et al. both reported an increased abundance of VIM on SAT of individuals with obesity [24, 25].

##### Proteins Related to Inflammation and Oxidative Stress

Chronic low-grade inflammation has been increasingly recognized to be involved in the pathophysiology of metabolic disease such as obesity [121]. Moreover, it is known that obesity promotes oxidative stress by producing oxidants and reducing antioxidant levels, contributing to the development of obesity-related complications [122]. Four proteins related to inflammation and oxidative stress have shown a consistent increase in subjects with obesityas compared to their normal-weight counterparts, while 2 proteins were found to be increased/decreased and only 1 protein was found to be consistent decreased in different studies.

##### Amyloid P-Component, Serum

Amyloid P-component, serum (APCS) is an acute phase protein made by the liver and secreted into the blood which regulates several aspects of the immune system [123]. Elevated levels of this protein in obesity have been reported, accompanied by a positive correlation with BMI and risk of cardiovascular diseases [124]. However, the role of APCS in human obesity has not been clearly elucidated. Studies may indicate that levels of this protein are elevated compared with non-obese controls but, in contrast, this may be a mechanism to down-regulate the effects of obesity, rather than a cause of obesity [125]. According to our results, APCS abundance have been found upregulated in plasma and EVs of individuals with obesity compared to lean controls, suggesting that this protein abundance may be the result of adaptation of the organism to the low-grade chronic inflammatory that underlies severe obesity [14, 46].

### Catalase

Catalase (CAT) is an essential antioxidant enzyme that protects cells against cellular toxic effects mediated by removing reactive oxygen species [126]. The antioxidant activity of CAT is significantly diminished in adults with obesity [122]. However, increased abundance of CAT, together with other proteins related to metabolic pathways has been associated with human AT protection and insulin-stimulated glucose uptake improvements in obesity [127]. Decreased levels of CAT were found in patients with morbid obesity after bariatric surgery [126]. Moreover, a significant decrease in abundance of CAT was found in patients with morbid obesity comparing to lean controls, additionally pointing to decrease in lipolysis within the mature adipocytes of SAT of this population [25]. In this regard, Kras et al. reported a decrease in CAT abundance in SKM proteome of subjects with obesity compared to controls [26]. Accordingly, upregulation of CAT may promote insulin sensibility and protect against obesity by influencing energy expenditure processes [25, 26].

### Complement C3

Complement C3 (C3) is part of the complement system, a complex enzymatic cascade consisting of more than 50 circulating and cell surface proteins working in cascades of stepwise protease activation, playing an important role as component of immune system [128]. C3 is a fundamental factor in metabolic organs and metabolic diseases, affecting insulin secretion and adipocyte maturation [129]. Moreover, previous studies have shown altered complement system in obesity, where excessive activation of the classic pathway of complement commonly occurs [130]. Increased C3 abundance was found increased in 2 independent studies of the present systematic review comparing EVs and plasma of individuals with obesity and lean controls and revealing the role of C3 in as an early marker for obesity and some related cardiovascular diseases [14, 46].

### Galectin-1

Galectin-1 (LGALS1) is a carbohydrate-binding protein that plays key immune regulatory roles in autoimmunity and chronic inflammation [131]. Moreover, it plays a role in a variety of cell functions including interferes with cell adhesion, proliferation, differentiation and angiogenesis [132]. LGALS1 is expressed in many tissues under normal and pathological conditions. Its abundance has been reported to be increased in obesity, both in the circulation and in the AT [133]. Moreover, LGALS1 expression has been reported to be decrease in participants with obesity during weight loss while increased during weight gain [133]. In this regard, LGALS1 was found increased on SAT of individuals with obesity in two different studies in the present systematic review [24, 25].

### Glutathione-S-Transferase P

Glutathione-*S*-transferase P (GSTP1) is an antioxidant enzyme involved in the formation of prostaglandins [134], with a catalytic detoxification role through inactivating byproducts of oxidative stress [135]. Previous studies have reported the association between GSP1 and some metabolic disorders [136]. Moreover, a positive correlation between GSTP1 polymorphism and obesity was observed on young adults with obesity, revealing its significant role in the increase of susceptibility of obesity and cardiovascular risk in this population [137]. Individuals who carry less efficient alleles of detoxification enzymes GSTP1 are subject to lower production or inefficient activity of these detoxification enzymes, which favours the development of obesity [137]. These findings are consistent with Boden et al. who reported upregulation of GSTP1 in VAT of patients with obesity compared to healthy controls [24]. In contrast, the abundance of GSTP1 was decreased in platelets from patients with obesity compared to individuals without obesity [55], which may also increase the production of oxidative agents and pro-inflammatory mediator in some cases [138].

### Lysozyme C

Lysozyme C (LYZ) is a component of the innate immune system that exerts anti-microbial effects through the hydrolysis of bacterial cell wall peptidoglycan [121]. It is considered as an important contributor to chronic low-grade inflammatory state. However, although it is important for driving a pro-inflammatory response, LYZ also plays a role in limiting inflammation system [139]. Decreased expression of LYZ was found in intestine of subjects with obesity [140]. Moreover, LYZ levels in plasma were significantly increased in obesity in direct link with obesity-associated metabolic disturbances and inflammatory parameters [121]. LYZ abundance was found decreased in circulating samples including platelets or plasma of patients with obesity compared to lean control [15, 46]. In contrast, a recent proteomics approach in obesity included in systematic review showed an increased protein abundance of LYZ in VAT of subjects with obesity compared to VAT of normoweight patients [43].

### Parkinson Disease Protein-7

Parkinson disease protein-7 (PARK7) is a multifunctional protein that has been described as a modulator of adipogenic differentiation and as a modulator of immune and inflammatory regulatory functions in many tissues [141]. Moreover, PARK7 has been reported to protect cells from oxidative stress injury. Animal studies have shown that raised PARK7 is correlated with obesity [142]. Conversely, PARK7 knockout mice had protection from diet to become obese. However, their inherent metabolic propensities and experimental outcomes towards obesity were influenced by strain differences, age, the effects of different high-fat diet composition and feeding period [142]. In addition, some other proteomics studies in obesity suggests that PARK7 are a proper reference standard in obesity studies based on VAT [143]. PARK7 protein abundance was found increased on SAT and SKM of individuals with obesity [25, 26].

#### Enrichment Analysis

**Fig. 2:**
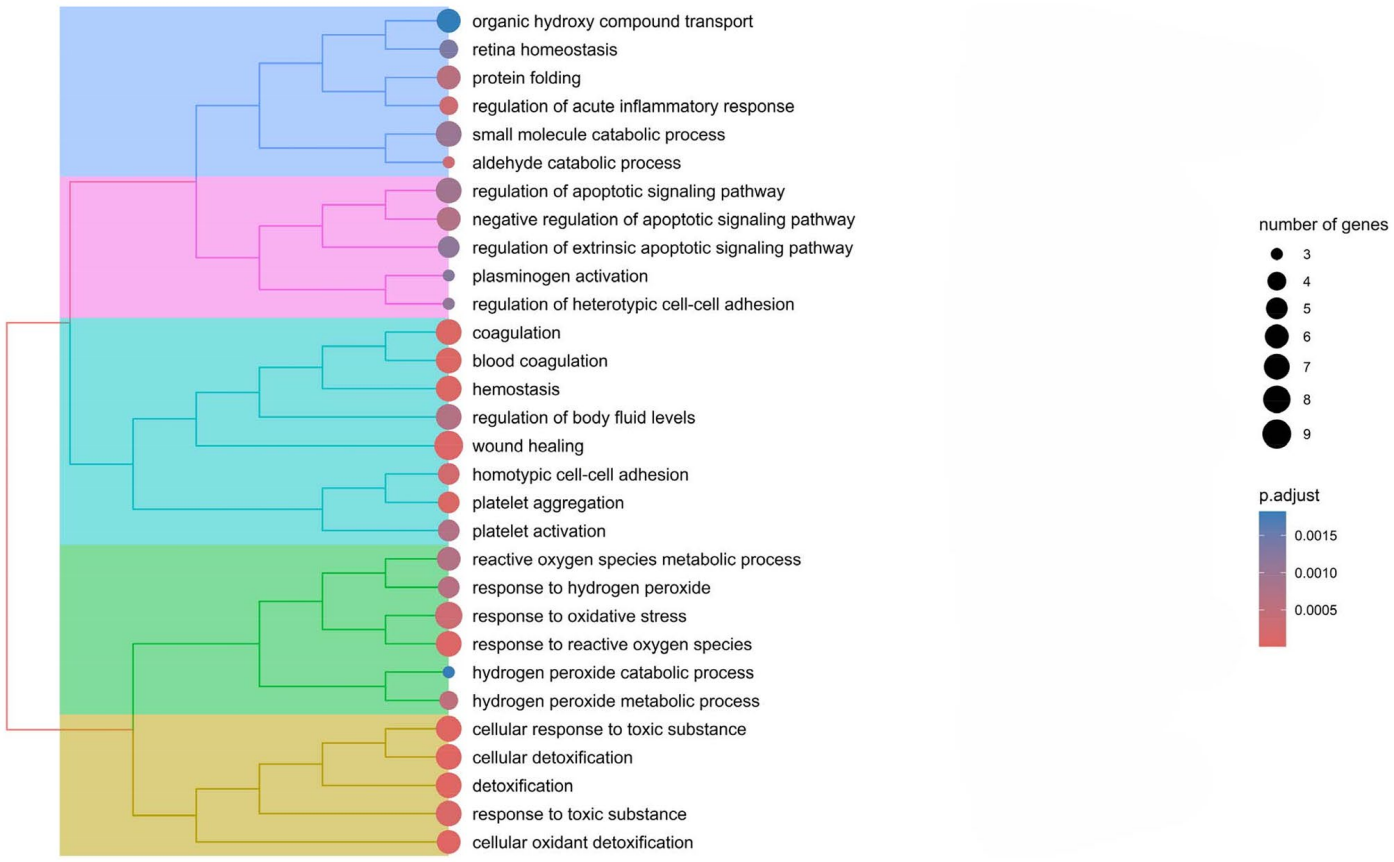
Enrichment analysis-GO: biological process

**Fig. 3:**
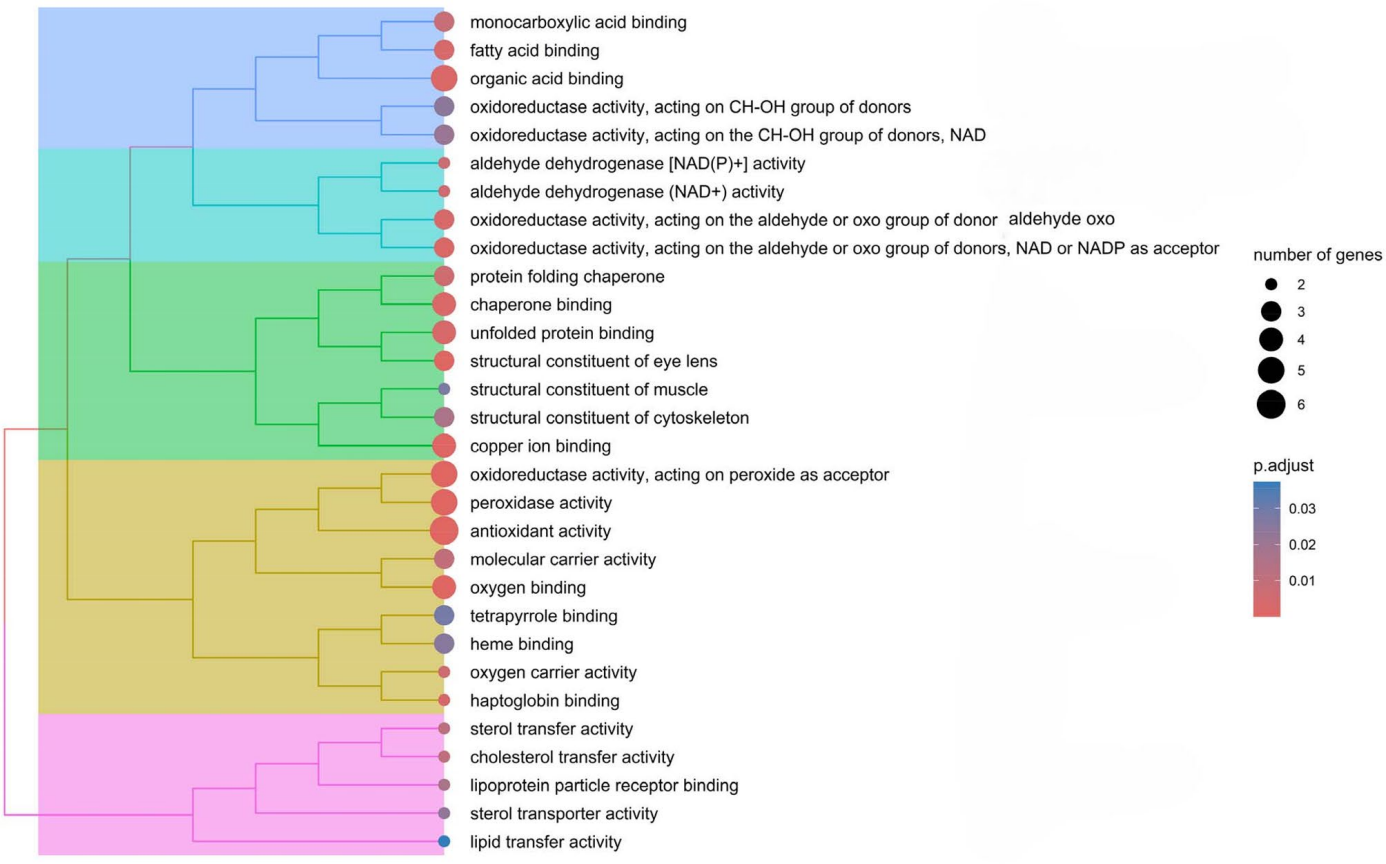
Enrichment analysis-GO: molecular functions altered in patients with obesity compared to normweight individuals

Databases resources Gene ontology (GO) and Kyoto Encyclopedia of Gene and Genomics (KEGG) were employed for the enrichment analysis of identified proteins in obesity. A systematic research of gene functions, linking genomic information with higher order functional information of proteins was conducted, including molecular function (MF), biological processes (BP) and cellular components (CC), as it is shown in online resource 2. The most significant results from the enrichment analysis of BP reveal that oxidative stress and haemostasis, including coagulation and platelet activation, were the most prevalent process (Fig. 2). Moreover, the molecular function enrichment analysis showed that the most relevant molecular functions were related to oxidative stress, metabolism and structural and protein folding (Fig. 3).

## Discussion

We have conducted an extensive systematic review of non-targeted proteomics studies in human obesity, identifying common trends in protein abundance profiles across various biological samples. A significant amount of knowledge has been gained in the last 2 decades on the proteomic profile of obesity. The application of proteomics represents a crucial approach in the management of obesity. This methodology has the potential to identify proteins involved in pathological processes and to evaluate changes in protein abundance during obesity development, contributing to the identification of early disease predisposition, monitoring the effectiveness of interventions and improving disease management overall. Additionally, it holds significant importance in drug development as proteomics identifies potential target molecules. Moreover, it can provide valuable insights into post-translational protein modifications, protein–protein interactions and signalling in obesity [144]. Proteomics can thus contribute significantly to our understanding of the complex interplay of proteins involved in obesity-related processes, offering potential targets for therapeutic interventions and personalized treatment strategies. The escalating global obesity epidemic represents one of the most serious public health challenges as the prevalence of obesity is increasing worldwide [145]. According to the World Health Organization (WHO), five million people die each year as a result of excess weight [https://www.who.int]. The alarming worldwide incidence increase of obesity is also associated with an array of metabolic pathologies, including T2DM and cardiovascular disease, spurring intense research efforts to understand the mechanisms underlying these disorders. Although the results of such efforts have led to the development of new treatment options, these conditions remain among the leading causes of global mortality and morbidity, emphasizing the need for more effective therapeutic and preventive measures. Existing trends indicate that the scope of the problem is only likely to grow, especially in rapidly developing parts of the world. Many contributing agents have now been identified, including genetic, dietary and environmental factors. However, the mechanisms by which excess nutrients and adiposity can ultimately result in one or more of a large cluster of chronic diseases are still being elucidated. Understanding the role of proteins in the onset and progression of obesity is crucial in medical research. This knowledge could significantly contribute to the effective management of obesity, reducing associated risks and improving overall health outcomes and quality of life of patients. It is essential to provide a fundamental basis for an accurate and comprehensive therapeutic approach to obesity. Such research can inform and enhance the efficacy of obesity management therapies. Our systematic review offers valuable insights into the proteomics field in obesity with significant implications for the understanding of protein regulation and biomarker discovery, and therefore contributing to open new pathways for personalized medicine of obesity.

**Fig. 4:**
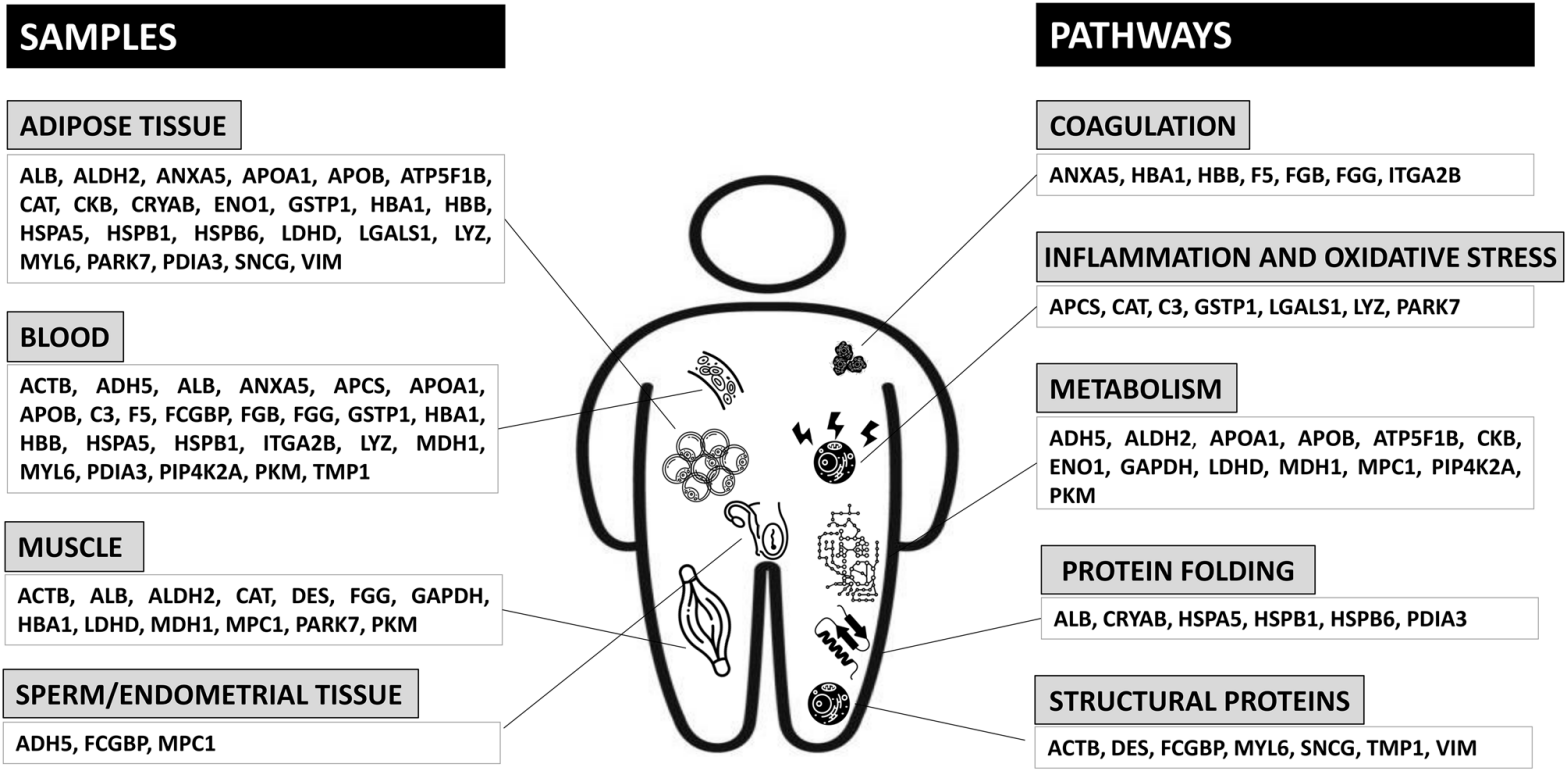
A summary of proteins altered in individuals with obesity compared to normweight patients

One of the primary achievements of this systematic review is the integration of proteomics data from multiple samples and analytical platforms. Our approach has involved harmonizing the data to identify consistent patterns in protein abundance and variation across different studies. Such integration provides a framework for future studies. In order to obtain more robust results, we focused on those proteins that were found in at least 2 proteomics studies. Figure 4 shows a comprehensive view of all the included proteins in the present systematic review, including functional processes mediated by them and human samples where have been found altered. Those proteins, forty-one, were related to several functions such as coagulation, inflammation/oxidative stress, metabolism, protein folding and structure/organization. All these processes have been reported to be altered in obesity.

Our analysis has revealed that the most altered proteins in obesity were predominantly involved in various aspects of metabolism, reflecting the intricate dysregulation of metabolic pathways associated with this condition. These findings are further supported by the enrichment analysis, which suggests an alteration in catabolic and metabolic processes in obesity (Figs. 2 and 3) and underscore the multifaceted nature of metabolic dysregulation in this condition [16]. Specifically, 13 proteins related to metabolic pathways were found altered in subjects with obesity. These proteins play crucial roles in maintaining energy homeostasis and facilitating ATP generation, essential processes for cellular function and overall energy balance [24, 25]. Other notable pathways affected were lipogenesis, the synthesis of fatty acids and triglycerides, which is altered in obesity due to excessive nutrient intake and adipose tissue expansion [51, 52]. Additionally, our research highlighted alterations in proteins involved in the metabolism and transport of lipoproteins, critical for lipid transport and cholesterol homeostasis [15, 25]. Proteins involved in gluconeogenesis, the synthesis of glucose from non-carbohydrate precursors, are also dysregulated in obesity, being the result or contributing to disturbances in glucose metabolism and insulin resistance in this pathology [51, 52]. Moreover, there are advances in pharmacological therapies targeting metabolic proteins. Studies have shown that some metabolic proteins could have a potential use as biomarkers of weight regain [146], as well as pharmacological therapy for metabolic diseases [147]. Therapies that modulate the activity of ATP synthase have been explored to treat metabolic disorders [148]. On the other hand, it has been studied the increase of creatine kinase B (CKB), known to promote thermogenesis, has been investigated as a potential approach to counteract obesity [41, 149]. Enzymes of gluconeogenesis and glycolysis (such as glucose-3-phosphate dehydrogenase or pyruvate kinase [PK]) have been considered as potential targets for the treatment of metabolic disorders such as T2DM [150]. One important drug, metformin, which is an inhibitor of one isoform of PK, has been widely used to counteract glucose levels in diabetic patients [151]. Furthermore, there are also therapies that modify the levels or functions of apolipoproteins with the aim of treating cardiovascular diseases and cholesterol-related metabolic disorders [152].

Oxidative stress constitutes a distinctive hallmark of obesity [153]. Our systematic review reveals several altered antioxidants, key proteins associated with oxidative stress, in the proteome of individuals with obesity. These proteins consistently display a decrease in antioxidant activity in most of the studies. It is well-known that obesity promotes oxidative stress by producing oxidants and reducing antioxidant levels, contributing to the development of obesity-related complications, such as cardiovascular disease, insulin resistance and metabolic syndrome, among others [122]. Moreover, the enrichment analysis showed that many of the proteins reported in the present review are also involved in oxidative stress in several processes, such as process and response to hydrogen peroxide and reactive oxygen species, and cellular oxidant detoxification (Figs. 2 and 3). Furthermore, obesity is considered as a chronic low-grade systemic inflammation associated with increased inflammatory markers [154]. This inflammation also contributes to associated alterations in obesity. We found 4 proteins involved in inflammation altered in obesity, where most of the studies reported an increased abundance and the enrichment analysis showed some proteins involved in the regulation of acute inflammatory response. Establishing an inflammatory phenotype could be useful in classifying individuals at risk. Cross-sectional studies have consistently demonstrated that anti-inflammatory nutrients are associated with lower levels of inflammatory markers [155] and pharmaceutical agents have targeted inflammatory pathways as potential therapeutic avenues for T2DM [155]. Numerous studies have evaluated the positive effect of antioxidant supplements in obesity and associated metabolic disease by improving glycaemic control and lipid profile, oxidative stress and inflammation [156]. Moreover, there are several antioxidant enzyme mimics currently under exploration, with some actively undergoing clinical trials [157].

Our analysis identified 6 proteins involved in protein folding that were altered in obesity and the enrichment analysis showed that protein folding was a prevalent function performed by the identified proteins. Protein folding is a process that usually takes place in the endoplasmic reticulum, eliciting proper forming of proteins [70], and actively participating in the protein homeostasis of the cells. It is considered as a vital cellular process because proteins must be correctly folded into specific, three-dimensional shapes in order to function correctly [158]. Unfolded or misfolded proteins contribute to the pathology of many diseases, such as obesity and cardiometabolic related disease [159]. Moreover, previous studies have reported strong correlations between chaperones with greater food intake or weight regain percentage [146, 160]. In addition, human and animal studies have shown that pharmaceutical chaperones, which are small molecules designed to stabilize the folding of proteins, improve insulin sensitivity in subjects with obesity [161]. Therefore, future studies on the six proteins involved in protein folding reported in this systematic review could contribute to elucidate mechanism of action in obesity development and to progress in the therapeutic avenue of it.

Several structural proteins have been reported to be altered in obesity. It is well-known that obesity is characterized by the induction of several tissue remodelling and, therefore, it is not surprising to find altered structural proteins in different tissues, such as AT and SKM from patients with obesity. Moreover, structural proteins were found altered in platelets. Obesity alters platelet number, morphology and activity [162], and altered structural proteins could be part of as a result of these processes. Platelets are a pivotal component of the physiologic haemostatic balance, which is also maintained through coagulation pathway. It has been found that regulating platelet function is beneficial. For individuals with obesity, exercising can help regulate platelet function and haemostasis. When engaged in moderate-intensity exercise, platelet aggregation and clotting factors are reduced, which can potentially decrease the risk of thrombosis [163]. The proteins described in this context could be used to design monitoring panels for platelet modulation. Furthermore, obesity exerts significant effects on the coagulation system. Seven proteins with pivotal roles in coagulation processes were altered in individuals with obesity. Derangements of blood coagulation has been reported previously in obesity several times [164], describing obesity as a promoter of coagulation. The enrichment analysis showed a high prevalence of proteins involved in haemostasis, coagulation and platelet aggregation and activation. As circulating proteins involved in haemostasis, coagulation and platelet aggregation are indicative of the underlying pathological mechanisms occurring in obesity, their concentrations could serve as potential biomarkers for stratifying the risk of comorbidities associated with obesity. Utilizing these proteins in predictive mathematical models may enable the development of risk stratification tools applicable in high-performance clinical settings.

**Fig. 5:**
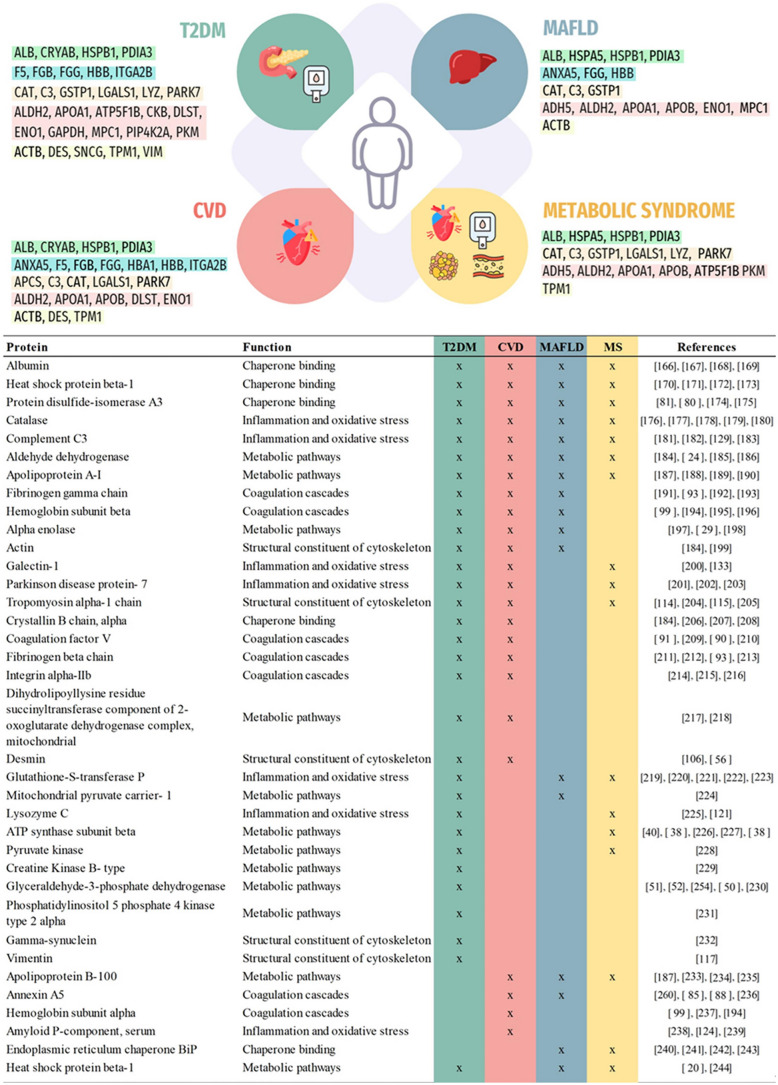
Relevant obesity proteomics biomarkers associated with obesity-related diseases. Colour-code represent their molecular function chaperon binding (green), coagulation cascades (light blue inflammation and oxidative stress (orange), metabolic pathways (red)) and structural constituent of cytoskeleton (yellow)

Many of the identified proteomic biomarkers of obesity have also been reported to be dysregulated in obesity-related disease, such as T2DM, MAFLD, CVD and metabolic syndrome, among others (Fig. 5). Seven proteins have been reported to be altered in the four selected obesity comorbidities. These proteins belong to metabolic pathways (aldehyde dehydrogenase X and apolipoprotein A1), the chaperone family (albumin, heat shock protein beta 1, protein disulfide-isomerase A3), and oxidative stress and inflammation proteins (catalase and complement C3). These proteins could potentially serve as biomarkers for the progression of obesity and the development of comorbidities, thereby contributing to personalized medicine within the field of obesity. In proteomics, relying on a single biomarker may not provide a complete understanding of the complexity of the picture. Furthermore, certain biomarkers consistently appear, indicating a significant potential beyond their individual roles and pointing to common pathways of metabolic dysfunction response. Developing comprehensive panels for analysing the progression of obesity and enabling early detection of its comorbidities is becoming increasingly crucial in biomedical research.

Our analysis has revealed substantial variations in protein profiles across various human samples, spotlighting how obesity impacts tissue function at the molecular level. Higher number of altered proteins were found in AT and blood (platelets, plasma or EVs) from patients with obesity and those proteins were involved in the 6 reported biological processes. In obesity, the most relevant tissue is the AT as obesity is the result of AT expansion. Moreover, adipose tissue becomes dysfunctional in obesity and compromise global metabolic and health homeostasis. Blood, the circulatory system, transport not only nutrients and by-products of cells but also transport mediators of intercellular communication. Therefore, both tissues are relevant in obesity and proteins found on them can be important to understand obesity pathology and can represent new treatment avenues.

The application of proteomics techniques in the study of obesity has limitations and presents significant challenges. Current challenges include the multifactorial pathophysiology of obesity, the standardisation operating conditions and procedures, the sample selection and sample size, the heterogeneity of the proteins detected based on protocols/platforms, the need for computational tools to assess the biological significance of detected proteins, the validation of proteomic findings and the translation of experimental data to clinical practice.

Obesity is influenced by genetic predispositions, environmental factors and lifestyle choices, making it challenging to attribute to specific proteins. Additionally, proteomic studies face challenges related to the type of sample and processing, the invasive nature of obtaining tissue samples and the limited availability of tissue specimens. Moreover, limitations in current proteomic technologies include the inability to detect proteins of very low abundance and quantify small-fold changes in abundance accurately. Different experimental platforms are used in proteomics, each with advantages and limitations. No single method can detect and identify the whole proteome, so different techniques should be seen as complementary, not exclusive. Bioinformatics plays a critical role in interpreting proteomic results, utilizing various techniques for managing, analysing and interpreting large datasets. Of note, only when submitted to appropriate bioinformatics tools, proteomic results serve to approach and solve biological problems. Finally, proteomics studies were limited by the possibility of a selection bias derived from the recruitment of both patients and controls in clinical-based population and by their relatively small sample sizes. Overcoming current limitations and advancing our understanding of obesity pathophysiology requires standardisation efforts, technological advancements and integration with other omics approaches. While we have made diligent efforts to account for these limitations, they remain potential sources of bias within the analysis. Therefore, future studies would benefit from standardized reporting and improved data sharing practices. Current standardisation efforts, such as the Human Proteome Organization (HUPO) Proteomics Initiative (PSI), aim to facilitate data comparison, exchange and verification of proteomics data. The development of more effective tools for data analysis and interpretation, and the improvements in the sensitivity of mass spectrometry instrumentation can help overcome the limitations and advance our understanding of obesity pathophysiology.

This systematic proteomics review presents several strengths that contribute to its significance in the field of obesity and associated metabolic diseases. First, the comprehensive nature of our analysis, selecting those altered proteins that were reported in at least 2 studies, encompassing a multitude of proteomic studies, allows us to draw conclusions about protein profiles across diverse human biological samples. This data integration enhances the robustness of our findings and provides a holistic perspective on the intricate interplay between proteins and the pathophysiology of obesity. Additionally, our incorporation of enrichment analyses reinforces the underlying molecular processes and metabolic pathways associated with obesity, further enriching our comprehension of the subject.

Proteomics offers a comprehensive overview of variations in protein abundance. One of the proteome’s strengths lies in its dynamic responsiveness to environmental stimuli, including dietary factors and chemical exposures. Moreover, due to translational processes and post-translational modifications, direct correlation with the transcriptome is not always observed. Therefore, proteomic approaches are one layer of omics information and must be complemented and integrated with data obtained from genomics, epigenomics, transcriptomics, metabolomics and metagenomics approaches, to unravel the complex molecular and cellular modifications resulting in obesity. Obesity is driven by a combination of an underlying genetic predisposition, and environmental factors. Genomics identifies genetic variations that may be associated with diseases and prognosis, while epigenomics studies the heritable changes and chemical modifications that occur in our DNA. Transcriptomics examines messenger RNAs (mRNAs) and non-coding RNA (ncRNAs) to understand gene expression, while metabolomics studies the small molecules involved in metabolic pathways to understand the biochemical processes. Human metagenomics examines the complete set of genes and genomes of the microbiota (bacteria, archaea, eukaryotes, and viruses) that reside in and on a person. Integrating multi-omics approaches in data analysis provides a more comprehensive view of molecular pathways underlying the development of obesity and comorbidities. It can lead to a significant shift from a generalized approach to a precise obesity management strategy. This includes precise prevention methods for obesity onset, tailored medicine for treating obesity, and targeted risk reduction strategies for preventing secondary diseases associated with obesity.

After the extensive research conducted in the proteomics analysis of individuals with obesity, the research faces a significant challenge in verifying the role of the identified proteins proposed in this systematic review. These proteins are being considered as potential indicators predicting susceptibility to obesity or its associated complications. Addressing this substantial challenge will require large cohorts comprising healthy and/or individuals with obesity to evaluate the significance of these proteins as biomarkers—a task not without considerable complexity.

The extensive research conducted in the proteomics analysis of individuals with obesity have shown proteins with significant potential as indicators for predicting susceptibility to obesity or its associated complications, enabling early detection screening, patient stratification, progression monitoring and identification of novel pharmaceutical targets for obesity and related diseases. Nevertheless, as the studies analysed in the present meta-analysis are cross-sectional, the research faces a significant challenge in verifying the causality and the role of the identified proteins proposed in this systematic review. Addressing this substantial challenge will require large prospective cohort studies comprising healthy and/or individuals with obesity to evaluate the significance of these proteins as biomarkers—a task not without considerable complexity. Therefore, future studies could focus on the evaluation of these proteins in subjects without obesity and observe if those with altered proteins are more prone to become obese. Similarly, a biomarker that elucidates the potential complications in patients with obesity, such as cardiovascular or hepatic diseases, is also of interest. To verify this, a cohort of individuals with obesity could be selected, examining those with additional complications and assessing whether these are associated with specific proteins. This could serve as a biomarker for obesity-related complications, some of which are challenging to diagnose early, like atherosclerosis before a heart attack or stroke. Alternatively, examining individuals with obesity without existing complications and monitoring the development of complications could provide insights into whether these proteins function as predictive biomarkers for obesity-related diseases. In addition, monitoring these identified proteins after obesity treatment, whether through surgery or nutritional pharmaceutical interventions, could provide physicians with real time functional insights regarding the efficacy of the administered treatments.

In conclusion, our systematic proteomics review represents a substantial step forward in unravelling the complexities of obesity-related protein changes, offering valuable insights into the pathophysiological mechanisms, unlocking potential avenues for biomarker discovery and personalized medicine. Several proteomic biomarkers of obesity involved in metabolic pathways, the chaperone family and oxidative stress and inflammation proteins have also been reported to be dysregulated in obesity-related disease, which could potentially serve as biomarkers for the progression of obesity and the development of comorbidities, contributing to personalized medicine in the field of obesity. However, it is crucial to emphasize the need for validation studies in larger patient cohorts to enhance the robustness of these findings to build a stronger basis for this research in obesity.

### Supplementary Information

Below is the link to the electronic supplementary material.

Supplementary file1 (DOCX 17 KB)

Supplementary file2 (XLSX 34 KB)

## Data Availability

No datasets were generated or analysed during the current study.
