# Correction: A Systematic Review of Proteomics in Obesity: Unpacking the Molecular Puzzle

**DOI:** 10.1007/s13679-024-00575-y

**Published:** 2024-05-24

**Authors:** Alba Rodriguez-Muñoz, Hanieh Motahari-Rad, Laura Martin-Chaves, Javier Benitez-Porres, Jorge Rodriguez-Capitan, Andrés Gonzalez-Jimenez, Maria Insenser, Francisco J. Tinahones, Mora Murri

**Affiliations:** 1 Endocrinology and Nutrition UGC, Hospital Universitario Virgen de La Victoria, Málaga, Spain; 2 Instituto de Investigación Biomédica de Málaga y Plataforma en Nanomedicina-IBIMA Plataforma BIONAND, Hospital Clínico Virgen de La Victoria, Málaga, Spain; 3 CIBER Fisiopatología de La Obesidad y Nutrición (CIBEROBN), Instituto de Salud Carlos III, Málaga, Spain; 4 Department of Molecular Genetics, Faculty of Biological Sciences, Tarbiat Modares University, Tehran, Iran; 5 Heart Area, Hospital Universitario Virgen de La Victoria, Instituto de Investigación Biomédica de Málaga y Plataforma en Nanomedicina-IBIMA Plataforma BIONAND, Malaga, Spain; 6 Department of Dermatology and Medicine, Faculty of Medicine, University of Malaga, Malaga, Spain; 7 Department of Human Physiology, Physical Education and Sport, Faculty of Medicine, University of Malaga, Malaga, Spain; 8 Biomedical Research Network Center for Cardiovascular Diseases (CIBERCV), Instituto de Salud Carlos III, Madrid, 28029 Spain; 9 ECAI Bioinformatic Institute of Biomedical Research of Malaga (IBIMA), Maria Insenser, Malaga, Spain; 10 Diabetes, Obesity and Human Reproduction Research Group, Department of Endocrinology & Nutrition, Hospital Universitario Ramón y Cajal & Universidad de Alcalá & Instituto Ramón y Cajal de Investigación Sanitaria (IRYCIS) & Centro de Investigación Biomédica en Red de Diabetes y Enfermedades Metabólicas Asociadas (CIBERDEM), Madrid, Spain

**Current Obesity Reports**

10.1007/s13679-024-00561-4

The original version of this article unfortunately contained an error in the title. The number “92” should be deleted. The correct article title is “A Systematic Review of Proteomics in Obesity: Unpacking the Molecular Puzzle”.

The original article has been corrected.

