# Supplementary material for: A Systematic Review of Proteomics in Obesity: Unpacking the Molecular Puzzle
Source: Curr Obes Rep. 2024 May 4;13(3):403–38. doi: 10.1007/s13679-024-00561-4 (PMC11306592; doi:10.1007/s13679-024-00561-4)
Supplement: Supplementary file 1 — Supplementary file1 (DOCX 17 KB) [file 13679_2024_561_MOESM1_ESM.docx]

**Annex 1**. Quality Assesment; Relationship between obesity and proteomics have been sistematically reviewed and selected articles match CASP Checklist

| **Author (year)** | **Are the results of the study valid?** | **What are the results?** | **Will the results help locally?** |
| --- | --- | --- | --- |
| **Barrachina *et al.* (2018)** | Yes | Relevance of plasma-derived-EVs proteins as a source of potential biomarkers for  the development of atherothrombotic events in obesity. | Yes |
| **Barrachina *et al.* (2019)** | Yes | Combination of proteomics and functional analyses could elucidate potential antiatherothrombotic targets in platelet-related diseases and obesity. | Yes |
| **Benabdelkamel *et al.* (2015)** | Yes | Overweight group expressed proteins were related to cell-to-cell signaling and interaction; in contrast, in the morbid obese group, the major interacting pathways are associated with lipid metabolism, small molecule biochemistry and cancer, providing insights into metabolic differences in obesity. | Yes |
| **Boden *et al.* (2008)** | Yes | Endoplasmic reticulum stress activation of JNK may be a link between obesity, insulin resistance, and inflammation. | Yes |
| **Giebelstein *et al.* (2012)** | Yes | Increased glycolytic and decreased mitochondrial protein abundance together with a shift in muscle properties towards a fast witch pattern in the absence of marked changes in fibre type distribution contribute to insulin resistance in obesity. | Yes |
| **Giuliani *et al.* (2022)** | Yes | Obesity was associated with significant endometrial proliferative phase proteomic differences, affecting the hormonal and immunologic pathways. | Yes |
| **Grande *et al.* (2019)** | Yes | MPs from obese individuals presented enhanced capacity to cause changes in the expression of EMT and EndMT marker genes and to induce COX-2, which might contribute to the increased risk for the development of thrombosis and multiple malignancies in obesity. | Yes |
| **Hittel et *al.* 2005** | Yes | AK1, aldolase A, and GAPDH proteins are increased in obese/overweight and morbidly obese SKM of women compared to lean control subjects. These changes may be compensated for the progressive decrease in muscle mitochondrial function in  obese individuals, which contributes to the loss of glucose and lipid homeostasis over time and to the eventual development of obesity-related diseases. | Yes |
| **Hwang *et al.* (2010)** | Yes | Reduction in mitochondrial proteins in insulin-resistant muscle suggest that changes in muscle structure, protein degradation and folding characterize insulin resistance and obesity. | Yes |
| **Karlsson *et al.* (2009)** | Yes | Obesity is associated with alterations in the LDL protein composition, affecting lipid metabolism in this condiction. | Yes |
| **Kras *et al.* (2018)** | Yes | Obesity was associated with differential effects on metabolic pathways linked to protein translation in the subsarcolemmal mitochondria and ATP formation in the intermyofibrillar mitochondria. | Yes |
| **Kriegel *et al.* (2009)** | Yes | Novel non-invasive experimental tool in the diagnosis of male infertility and in monitoring any fertility-restoring therapy. | Yes |
| **Oberbach *et al.* (2011)** | Yes | Complement system is related to obesity. Novel proteins (C3b, CLU, VDBP) and metabolites are identified and previously discovered markers (PEDF, RBP4, C3, ATIII, and SAP) of body fat mass changes are confirmed. | Yes |
| **Pini *et al.* (2020)** | Yes | Oxidative stress and inflammation are closely tied to reproductive dysfunction in obese men. | Yes |
| **Shang *et al.* (2019)** | Yes | Differential identified proteins could be potential candidates in addressing the role of VAT in the development of obesity. | Yes |
| **Si *et al.* (2021)** | Yes | In obese subjects, mitochondria from GCs were damaged and the endoplasmic reticulum stress response was accompanied by dysregulated hormonal synthesis whereas none of these changes occurred in normal-weight subjects. | Yes |
